# Alterations of SIRT1/SIRT3 subcellular distribution in aging undermine cardiometabolic homeostasis during ischemia and reperfusion

**DOI:** 10.1111/acel.13930

**Published:** 2023-08-03

**Authors:** Jingwen Zhang, Hao Wang, Lily Slotabec, Feng Cheng, Yi Tan, Ji Li

**Affiliations:** ^1^ Department of Physiology and Biophysics University of Mississippi Medical Center Jackson Mississippi USA; ^2^ Department of Surgery University of South Florida Tampa Florida USA; ^3^ Department of Pharmaceutical Sciences, College of Pharmacy University of South Florida Tampa Florida USA; ^4^ Pediatric Research Institute, Department of Pediatrics University of Louisville Louisville Kentucky USA; ^5^ G.V. (Sonny) Montgomery VA Medical Center Jackson Mississippi USA

**Keywords:** aging, fatty acid oxidation, ischemia/reperfusion, SIRT1, SIRT3

## Abstract

Age‐related sensors Sirtuin1 (SIRT1) and Sirtuin3 (SIRT3) play an essential role in the protective response upon myocardial ischemia and/or reperfusion (I/R). However, the subcellular localization and co‐regulatory network between cardiac SIRT1 and SIRT3 remain unknown, especially their effects on age‐related metabolic regulation during acute ischemia and I/R. Here, we found that defects of cardiac SIRT1 or SIRT3 with aging result in an exacerbated cardiac physiological structural and functional deterioration after acute ischemic stress and failed recovery through reperfusion operation. In aged hearts, SIRT1 translocated into mitochondria and recruited more mitochondria SIRT3 to enhance their interaction during acute ischemia, acting as adaptive protection for the aging hearts from further mitochondria dysfunction. Subsequently, SIRT3‐targeted proteomics revealed that SIRT1 plays a crucial role in maintaining mitochondrial integrity through SIRT3‐mediated substrate metabolism during acute ischemic and I/R stress. Although the loss of SIRT1/SIRT3 led to a compromised PGC‐1α/PPARα‐mediated transcriptional control of fatty acid oxidation in response to acute ischemia and I/R, their crosstalk in mitochondria plays a more important role in the aging heart during acute ischemia. However, the increased mitochondria SIRT1‐SIRT3 interaction promoted adaptive protection to aging‐related fatty acid metabolic disorder via deacetylation of long‐chain acyl CoA dehydrogenase (LCAD) during ischemic insults. Therefore, the dynamic network of SIRT1/SIRT3 acts as a mediator that regulates adaptive metabolic response to improve the tolerance of aged hearts to ischemic insults, which will facilitate investigation into the role of SIRT1/SIRT3 in age‐related ischemic heart disease.

## Graphical abstract

The dynamic network of SIRT1/SIRT3 in response to myocardial ischemia and reperfusion stress.

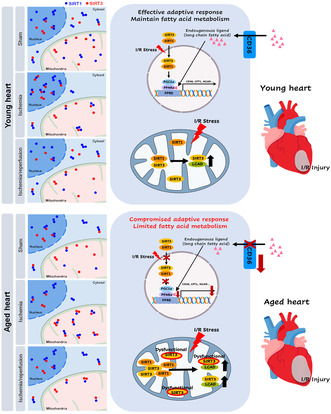

**Abbreviations**
CVD: cardiovascular disease
CPT1β: carnitine palmitoyltransferase 1β
IHD: ischemic heart disease
I/R: ischemia and reperfusion
LCAD: long‐chain acyl‐CoA dehydrogenase
MCAD: medium‐chain acyl‐CoA dehydrogenases
NAD^+^
: nicotinamide adenine dinucleotide
OXPHOS: oxidative phosphorylation
PPARα: peroxisome proliferator activated receptor alpha
PGC‐1α: PPAR gamma coactivator 1‐alpha (PGC‐1α)
PDH E1α: pyruvate dehydrogenase E1α (PDH E1α)
Sirtuins: silent information regulators
SCAD: short‐chain acyl‐CoA dehydrogenases.
TCA: tricarboxylic acid
TEM: transmission electron microscopy
VLCAD: very long‐chain acyl‐CoA dehydrogenases

## 1 INTRODUCTION

Ischemic heart disease (IHD) accounts for the most serious cardiac issues (Akhtar, 2006; Benjamin et al., 2019). Compared to adult hearts, IHD carries the greatest burden for the older population (Chen et al., 2022). Numerous structural and functional alterations during aging render the heart more vulnerable to various stressors and culminate in the increasing risk of developing IHD (Christoffersen et al., 2014; Madhavan et al., 2018). Fatty acid β‐oxidation in mitochondria is a major energy source for the myocardium (Fukushima & Lopaschuk, 2016b) and the aged heart exhibits impaired metabolic flexibility caused by impaired mitochondrial oxidative phosphorylation (OXPHOS) (Lesnefsky et al., 2016). Changes in transcriptional and posttranslational control of fatty acid oxidative enzymes are the potential mechanism contributing to the ischemia‐induced shift in cardiac energy metabolism (Fukushima & Lopaschuk, 2016a; Rosano et al., 2008).

The mammalian silent information regulators (sirtuins) are a family of nicotinamide adenine dinucleotide (NAD^+^)‐dependent deacetylases that are closely related to the extension of lifespan (Imai & Guarente, 2014). Moreover, the absence of mammalian sirtuins plays an essential role in multiple crucial cellular processes to combat myocardial ischemia/reperfusion (I/R), including cell survival, DNA repair, inflammation, and metabolism (Zhang et al., 2020). These modifications controlled by sirtuins are associated with the transcriptional regulation of related genes and lysine acetylation of critical enzymes. In addition, the sirtuin family members localize in distinct subcellular compartments (Kwon et al., 2017; Tong et al., 2013) and appear to be changed in a tissue‐specific manner in some stress situations (Bao et al., 2010; Iwahara et al., 2012; Parodi‐Rullan et al., 2018).

Sirtuin1 (SIRT1) is the closest mammalian homolog to the yeast Sir2 protein in sequence and is expressed in a wide range of tissues such as the heart, liver, and muscle of mice (Imai & Guarente, 2010; Lavu et al., 2008). The nucleocytoplasmic shuttling of SIRT1 during cardiac development and physiological or pathological stimuli affects different deacetylated targets and transcription factors, further influencing its function. SIRT1 is exclusively expressed in the nucleus in the cardiomyocytes of mouse embryos but expressed in both the cytoplasm and nucleus in the adult heart (Tanno et al., 2007). Our previous research showed compromised SIRT1 nuclear shuttling in the aged hearts, which was worsened under ischemia and I/R stress (Tong et al., 2013). Moreover, the activation of SIRT1 in the young hearts upon acute ischemia and short‐time I/R stress failed in the aged upon ischemia. (Tong et al., 2013). However, the nucleocytoplasmic shuttling pattern of SIRT1 and its role in age‐related cardiac impaired metabolic flexibility during acute ischemia and long‐term I/R stress remain unclear.

The huge impact of Sirtuin3 (SIRT3) in the regulation of lifespan, cardiac function, and mitochondrial biology has been evaluated in numerous studies (Alrob et al., 2014; Hirschey et al., 2010; Koentges et al., 2015; Sundaresan et al., 2008). In mice, loss of SIRT3 in the aging heart results in decreased intolerance to I/R stress (Parodi‐Rullan et al., 2017). However, the different subcellular localization of SIRT3 and its activity has been a subject of considerable debate. Human SIRT3 (hSIRT3) was located in the nucleus with its unique N‐terminal mitochondrial localization sequence (MLS) and translocated into mitochondria upon cellular stress (Iwahara et al., 2012; Scher et al., 2007). These studies were opposite to Cooper and Spelbrink, who demonstrated that endogenous hSIRT3 was expressed predominately in the mitochondria, while overexpression of hSIRT3 lacking MLS resulted in the expression both in the cytoplasm and nucleus. Despite the loss of the N‐terminus compared to hSIRT3, the mouse SIRT3 (mSIRT3) still had two forms (Pillai et al., 2010). Both were enzymatically active and detected in the nuclear and cytoplasmic, and mitochondria fractions of H9cC2 cells, while their abundance in each fraction was disparate (Bao et al., 2010). In addition, a previous study showed localization of SIRT3 protein change from mitochondria to the nucleus is associated with the co‐expression with sirtuin (SIRT5). Interestingly, it has been recognized that liver SIRT1 mediated the SIRT3 activity via deacetylation during the obesity and aging process (Kwon et al., 2017). Till now, the subcellular localization, activity, and co‐regulatory network of cardiac SIRT3 are still uncertain, especially their effects on age‐related metabolic regulation during acute ischemia and long‐term I/R stress.

The present study investigated the altered distribution of short‐form SIRT1 and SIRT3 in young and aged hearts during acute ischemia and long‐term I/R stress. We attempted to elucidate the interaction between SIRT1 and SIRT3 under physiological and pathological conditions, as well as explored their effects on maintaining cardiac adaptive metabolic response. Our study demonstrated that age‐related SIRT1 and SIRT3 deficiency contributed to the mitochondrial metabolic homeostasis out of balance in the heart, resulting in exacerbated myocardial cell death and cardiac dysfunction upon acute ischemia stress, and cannot be recovered after 6 h reperfusion treatment. Aging‐related SIRT1 inactivation during ischemia led to an adaptive response of SIRT1 mitochondria relocation rather than nuclear. Our results provide novel mechanistic insights into the roles of the SIRT1/ SIRT3 subcellular regulatory network in protecting against cardiac acute ischemia and the recovery from I/R operation in the aging heart.

## 2 RESULTS

### 2.1 SIRT1 and SIRT3 deficiency with aging causes cardiac histological damage and augments cardiac sensitivity to ischemic insults

**FIGURE 1:**
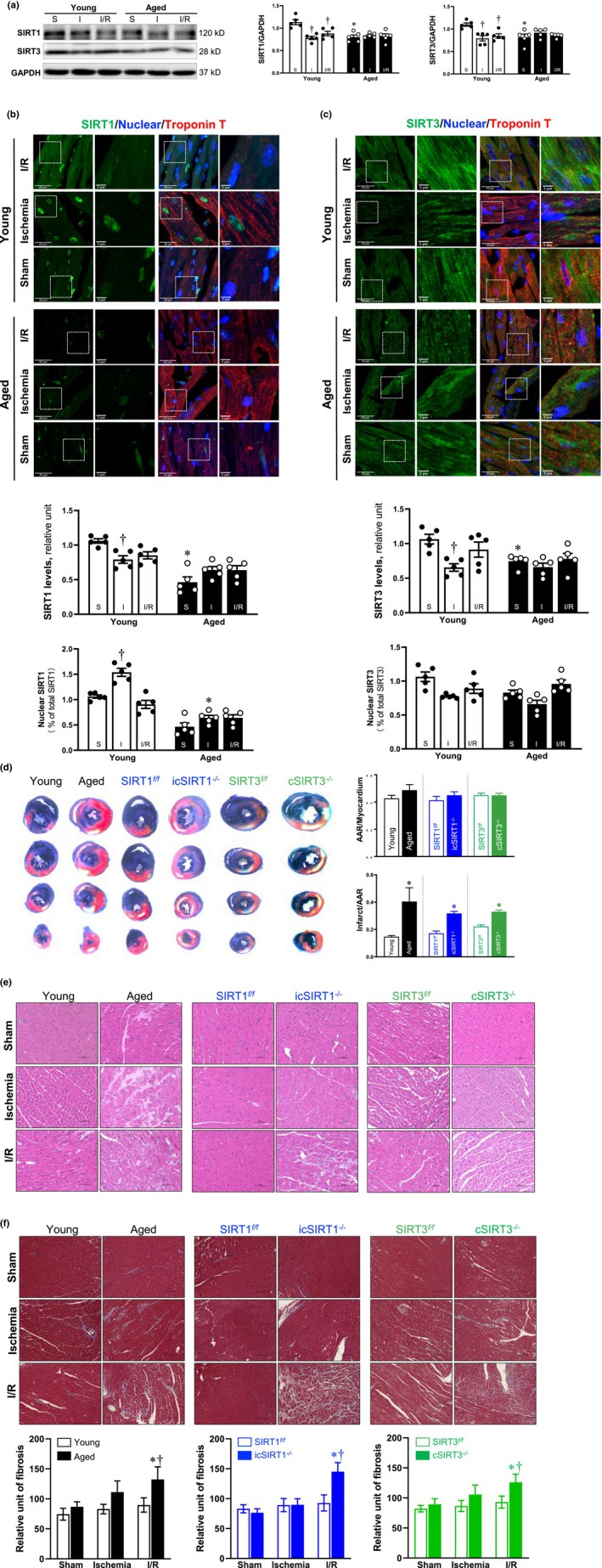
Deficiency of SIRT1 and SIRT3 with aging leads to cardiac vulnerable to acute ischemia and I/R stress. (a) The protein levels of SIRT1 and SIRT3 from the left ventricle of the male mouse hearts declined in aging, and a blunted response occurred in aged hearts during acute ischemia and I/R stress. (*N* = 5, values are mean ± SEM from five biological replicates, *p < 0.05 vs. young; ^†^ *p* < 0.05 vs. sham, respectively, two‐way ANOVA with Tukey's post hoc test). (b) and (c) Upper: Representative immunofluorescence staining images of SIRT1, SIRT3, troponin T, and DAPI in the area at risk (AAR) of young and aged male heart's left ventricle under sham, acute ischemia, or I/R conditions. Lower: Statistical analysis of SIRT1, SIRT3 staining and percentage of nuclear SIRT1 and SIRT3 in young and aged AAR under sham, acute ischemia, and I/R conditions. (*N* = 5, values are mean ± SEM from five biological replicates, *p < 0.05 vs. young; ^†^ *p* < 0.05 versus sham, respectively, two‐way ANOVA with Tukey's post hoc test). (d) Young (4–6 months)/aged (24–26 months) wild‐type C57BL/6J, SIRT1^*f/f*^, icSIRT1^*−/−*^, SIRT3^*f/f*^ and cSIRT3^*−/−*^ C57BL/6J male mice were subjected to in vivo regional acute ischemia for 30 min only or followed by 6 h of reperfusion. Left: Representative sections of the extent of myocardial infarction were presented. TTC staining showed a larger infarct area in aged versus young, icSIRT1^*−/−*^ versus SIRT1^*f/f*^, and cSIRT3^*−/−*^ versus SIRT3^*f/f*^ hearts, respectively. Right: The ratio of the AAR to the total myocardial area refers to the area affected by ischemia, and the ratio of the infarcted area to AAR is used to access the myocardium injury. (*N* > =3, values are means ± SEM from at least three biological replicates, *p < 0.05 versus young, SIRT1^*f/f*^, SIRT3^*f/f*^, respectively, two‐way ANOVA with Tukey's post hoc test). (e) Representative H&E‐stained myocardium of young (4–6 months), aged (24–26 months), SIRT1^*f/f*^, icSIRT1^*−/−*^, SIRT3^*f/f*^, and cSIRT3^*−/−*^ male mice hearts sham, acute ischemia, and I/R conditions. (f) Upper: Representative images of myocardial fibrosis measured by Masson's trichrome staining in young, aged, SIRT1^*f/f*^, icSIRT1^*−/−*^, SIRT3^*f/f*^, and cSIRT3^*−/−*^ hearts under sham, acute ischemia, and I/R conditions. Lower: Quantification analysis of Masson's trichrome staining. (*N* = 5, values are mean ± SEM from five biological replicates, *p < 0.05 versus sham, respectively: ^†^ *p* < 0.05 versus young I/R, SIRT1^*f/f*^ I/R, SIRT3^*f/f*^ I/R, respectively, two‐way ANOVA with Tukey's post hoc test).

To investigate the critical role of SIRT1 and SIRT3 in age‐related ischemic heart disease, the protein expression of SIRT1 and SIRT3 was first detected in young and aged mouse heart's left ventricle under sham operations, myocardial acute ischemia of 30 min and 30 min ischemia followed by 6 h reperfusion. The results demonstrated that SIRT1 and SIRT3 protein levels are decreased with aging, and both were downregulated in response to acute ischemia and I/R stress in the young left ventricle (Figure 1a). The confirmatory results were obtained by analyzing SIRT1 and SIRT3 protein expression levels in the area at risk (AAR) of young and aged mice heart's left ventricle by immunofluorescent staining (Shihan et al., 2021). Lower levels of SIRT1 and SIRT3 proteins were observed in aged AAR as compared to young AAR under sham conditions (Figure 1b,c). In addition, acute ischemic stress significantly decreased SIRT1 and SIRT3 total protein levels and enhanced the nuclear translocation of SIRT1 in young AAR, whereas blunted in aged AAR (Figure 1b,c). However, there is no significant change after I/R treatment in both young and aged AAR. These results indicate that cardiac SIRT1 and SIRT3 are decreased with aging, and there could be differences in the SIRT1 and SIRT3 expression between AAR and infarct area.

To assess the effects of age‐related deficiency in SIRT1 and SIRT3 on cardiac histology, mouse hearts were subjected to TTC and Evens Blue staining, HE, and Masson staining. The myocardial infarction measurements demonstrated that the infarct size was significantly larger in aged hearts as compared to young hearts after I/R surgery (Figure 1d). The deletion of cardiomyocyte SIRT1 (icSIRT1^−/−^) versus SIRT1^*f/f*^ mice also showed a larger infarction size after myocardial I/R stress (Figure 1d). Similarly, cSIRT3^*−/−*^ versus SIRT3^*f/f*^ mouse hearts are more sensitive to I/R stress as shown with larger myocardial infarction size (Figure 1d). Thus, age‐related absence of cardiac SIRT1/SIRT3 could be a factor causing vulnerability of the heart to ischemic insults. Moreover, the histopathologic evaluation demonstrated that the cardiomyocytes in young sham groups were orderly arranged, and dense, and the cardiac muscle fascicles were complete in shape. However, aged hearts are characterized by loss of cardiac cross striations and decreased cardiomyocyte cytoplasmic eosinophilia versus young hearts under sham conditions (Figure 1e). And there are more myofiber waviness, interstitial edema, and microvessels congested with sickled red blood cells (RBCs) in cardiomyocytes of aged versus young mice under acute ischemia and I/R conditions (Figure 1e). The cardiomyocytes showed slight disorder in arrangement and shape in icSIRT1^*−/−*^ versus SIRT1^*f/f*^ hearts under physiological conditions. Nevertheless, the icSIRT1^*−/−*^ showed disrupted cardiomyocytes with more severe fibrosis, occasional vacuolar degeneration, sickled RBCs, and necrotic debris after acute ischemia and I/R stress compared to SIRT1^*f/f*^ hearts (Figure 1e). The cardiomyocytes were orderly arranged and complete in shape in both cSIRT3^*−/−*^ and SIRT3^*f/f*^ sham hearts. While the myocytes of cSIRT3^*−/−*^ mice entered the irreversible state exhibiting small breaks and an increased degree of disorganized myocardium under acute ischemia and I/R stress in comparison to SIRT3^*f/f*^ mice (Figure 1e). It suggests that SIRT1 and SIRT3 play important roles in preserving myocardium structure during myocardial acute ischemia and I/R stress. Furthermore, substantial increases in perivascular and interstitial fibrosis in aged hearts under I/R treatment compared to the young I/R group (Figure 1f). Cardiomyocyte deficiency of SIRT1 or SIRT3 also showed increased cardiac fibrosis under I/R stress as compared to their flox littermates, respectively (Figure 1f). Thus, the results indicate that the deficiency of SIRT1 or SIRT3 in cardiomyocytes demonstrated aging‐like fibrosis in response to pathological conditions.

### 2.2 SIRT1 and SIRT3 deficiency with aging causes different alterations in IFM, SSM, and PNM during acute ischemia and I/R stress

**FIGURE 2:**
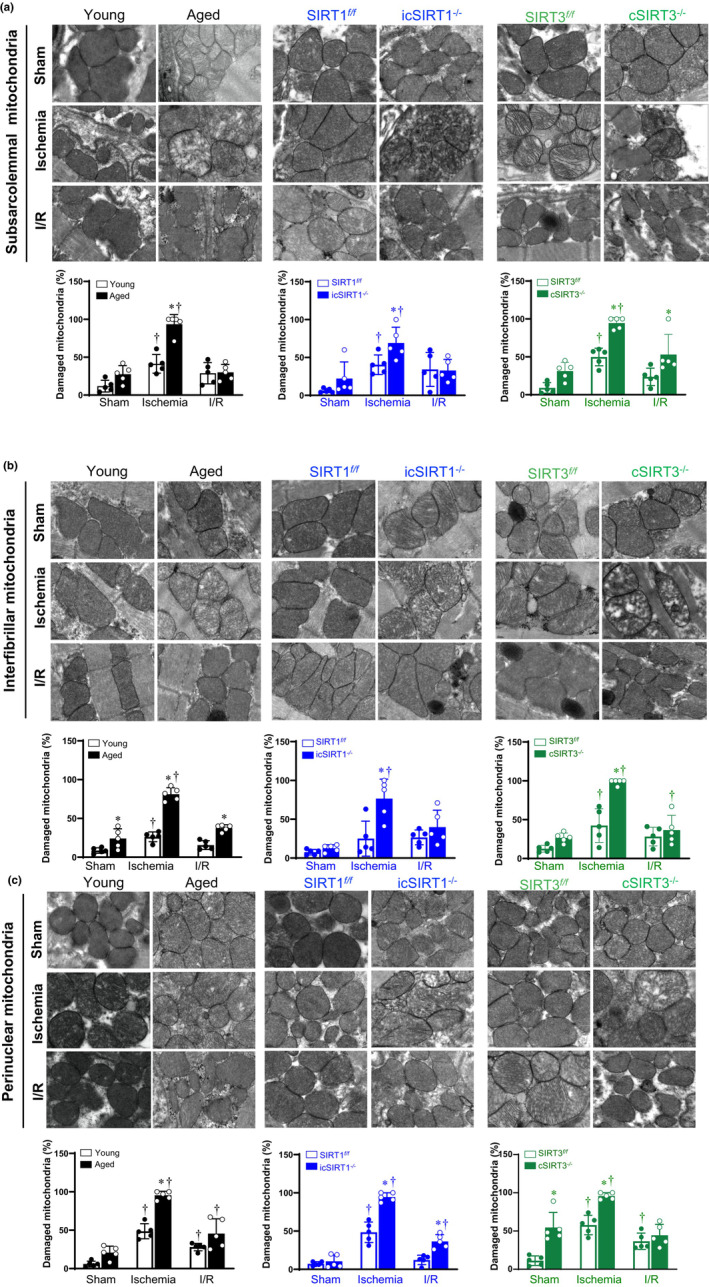
The morphological alterations in different cardiac mitochondria after acute ischemia and I/R operations. The transmission electronic microscope (TEM) images were obtained from subsarcolemmal mitochondria (a), interfibrillar mitochondria (b), and perinuclear mitochondria (c) in the left ventricle of young/aged C57BL/6J wild type, SIRT1^*f/f*^/icSIRT1^*−/−*^, SIRT3^*f/f*^/cSIRT3^*−/−*^ hearts under sham, acute ischemia, and I/R stress. Upper: Representative TEM at a magnification of 60,000; Lower: Quantitative analysis by ImageJ to calculate the percentage of damaged mitochondria (Value are means ± SEM from two biological replicates with five technical repeats at a magnification of 20,000. *p < 0.05 versus young, SIRT1^*f/f*^, SIRT3^*f/f*^, respectively; ^†^ *p* < 0.05 versus sham, respectively, two‐way ANOVA with Tukey's post hoc test).

We further examined the effects of cardiomyocyte SIRT1 and SIRT3 deletion on the mitochondrial structure of the AAR in hearts' left ventricles under sham operation, acute ischemia, and I/R stress. The transmission electronic microscope (TEM) was performed to examine the alterations in the ultrastructure of the perinuclear mitochondria (PNM), interfibrillar mitochondria (IFM), and subsarcolemmal mitochondria (SSM). The mitochondrial damage is characterized by swollen, homogenized, and whirled‐in cristae and disrupted outer membranes. As for SSM, the aged, icSIRT1^*−/−*^ and cSIRT3^*−/−*^ AAR showed significant damage in cristae under acute ischemic stress versus young, SIRT1^*f/f*^ and SIRT3^*f/f*^ AAR, respectively (Figure 2a). Similar observations occurred in both IFN (Figure 2b) and PNM (Figure 2c). All the SSM, IFM, and PNM were significantly damaged after the acute ischemic operation in the young AAR, which was further aggravated in the aged group. Notably, only IFM were significantly damaged after I/R stress in the aged AAR verse young group. The deficiency of cardiac SIRT1 and SIRT3 also showed aging‐like dramatic damage in the SSM, IFM, and PNM after the acute ischemic operation versus SIRT1^*f/f*^ and SIRT3^*f/f*^, respectively. Only PNM were significantly damaged after I/R stress in icSIRT1^*−/−*^ AAR verse SIRT1^*f/f*^ group, while only SSM were significantly damaged after I/R stress in cSIRT3^*−/−*^ AAR verse SIRT3^*f/f*^ group. Taken together, these data indicate that the deficiency of SIRT1 and SIRT3 with aging can cause alterations in mitochondrial morphology in response to acute ischemia. However, due to the distinct subcellular locales (Hollander et al., 2014), the three mitochondrial have different work in regulating cardiac mitochondrial homeostasis upon I/R stress. The absence of cardiac SIRT1 seems to have a greater effect on PNM during I/R treatment, while the absence of cardiac SIRT3 seems to have a greater effect on SSM.

### 2.3 Acute ischemia stress affects the subcellular distribution and SIRT1–SIRT3 interaction

**FIGURE 3:**
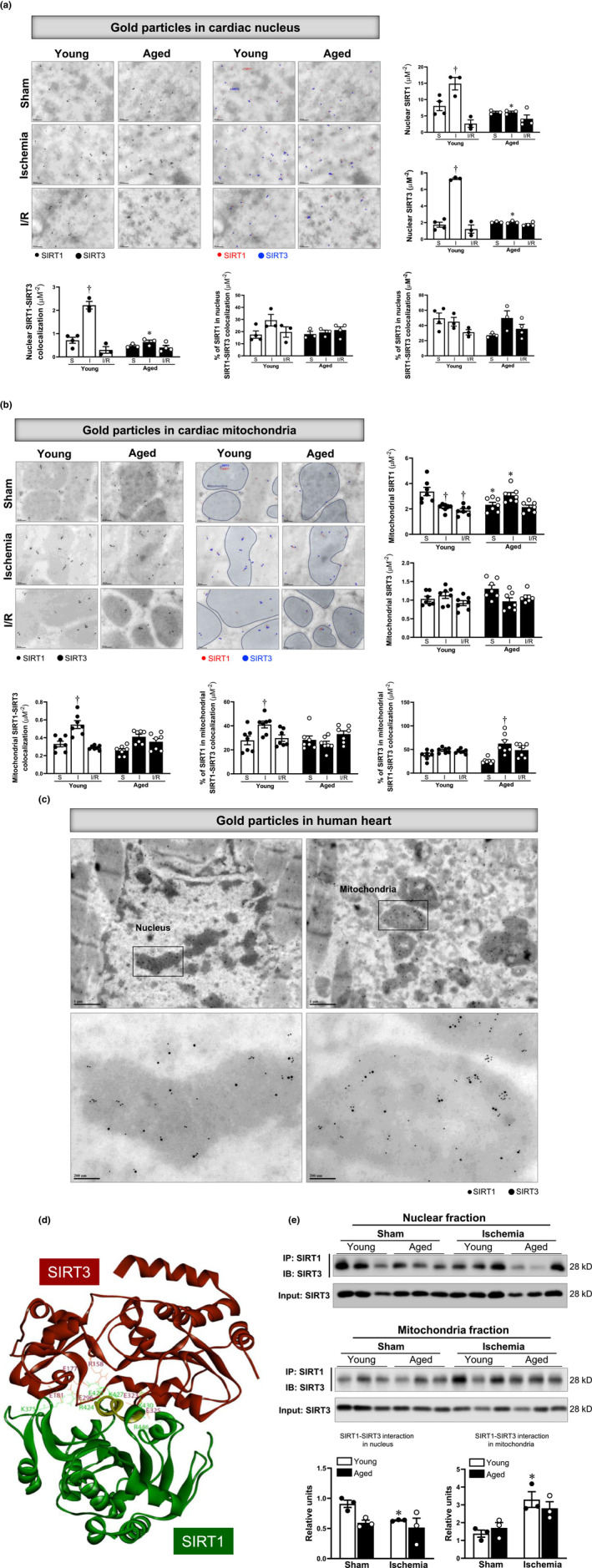
Distinct subcellular distribution and interaction of SIRT1 and SIRT3 with cardiac aging after acute ischemia and I/R operations. (a) Immunogold double labeling of SIRT1 and SIRT3 in the nucleus of the left ventricle AAR of young and aged hearts under sham, acute ischemia, and I/R conditions. Upper: Left, representative immunogold labeling images of SIRT1 (labeled with red dots) and SIRT3 (labeled with blue dots) in each group at a magnification of 100,000. Right, the distribution of nuclear SIRT1 and SIRT3 gold particles in the heart's left ventricle AAR. Lower: the colocalization of SIRT1 and SIRT3 in the myocardium nucleus and their percentage ratio involved in the colocalization. (*N* > =3 nucleus of each replicate, values are means ± SEM form at least three technical replicates at a magnification of 20,000. *p < 0.05 vs. young; ^†^ *p* < 0.05 vs. sham, respectively, two‐way ANOVA with Tukey's post hoc test). (b) Immunogold double labeling of SIRT1 and SIRT3 in the mitochondria of the left ventricle AAR of young and aged hearts under sham, acute ischemia, and I/R conditions. Upper: Left, representative immunogold labeling images of SIRT1 (labeled with red dots) and SIRT3 (labeled with blue dots) in each group at a magnification of 60,000. Right, the distribution of mitochondrial SIRT1 and SIRT3 gold particles in the heart's left ventricle AAR. Lower: the colocalization of SIRT1 and SIRT3 in the myocardium mitochondria and their percentage ratio involved in the colocalization. (*N* > =10 mitochondria of each replicate, values are means ± SEM from seven technical replicates at a magnification of 20,000. *p < 0.05 vs. young; ^†^ *p* < 0.05 vs. sham, respectively, two‐way ANOVA with Tukey's post hoc test). (c) Representative immunogold double labeling images of SIRT1 and SIRT3 at a magnification of 100,000 in the left ventricle of the human heart. (d) The protein–protein docking analysis indicated that SIRT1 and SIRT3 can form strong interactions. (e) The interaction of SIRT1 and SIRT3 in nuclear and mitochondrial portions of the left ventricle of young and aged hearts under sham or acute ischemia. (*N* = 3, values are means ± SEM from three biological replicates, *p < 0.05 vs. young; ^†^ *p* < 0.05 vs. sham, respectively, two‐way ANOVA with Tukey's post hoc test).

To verify the difference between SIRT1 and SIRT3 localization in cardiomyocytes under physiological and pathological conditions, the immunogold double labeling of the AAR in hearts' left ventricle from young and aged C57BL/6J mice was performed (Enger, 2017). In the nucleus, positive staining showed that there was an increased distribution of SIRT1 and SIRT3 in response to acute ischemic stress in young AAR, whereas this response was blunted in aged AAR (Figure 3a). The nuclear SIRT1–SIRT3 colocalization followed the same pattern, which was significantly increased in young AAR upon acute ischemic stress, and significantly decreased in aged AAR (Figure 3a). Moreover, although the abundance of SIRT1 or SIRT3 in their nuclear interaction have no significant changes under those stress, a light increase pattern of SIRT1 in young AAR and SIRT3 in Aged AAR under acute ischemia was observed. These results suggest the increased nuclear shuttling of SIRT1 increases its activity (Tong et al., 2013) and results in the enhanced colocalization between SIRT1 and SIRT3. These changes served as an adaptive response under ischemic conditions in young AAR, and this adaptive response is alleviated in aged AAR.

In response to both acute ischemia and I/R stress, the mitochondrial SIRT1 was reduced versus sham conditions in young AAR. Interestingly, mitochondrial SIRT1 decreased in aged AAR under sham conditions versus the young group, while there were more SIRT1 translocated into mitochondria in aged hearts upon acute ischemia (Figure 3b). The mitochondria SIRT1‐SIRT3 colocalization was significantly increased in young AAR upon acute ischemic stress, while blunted in aged AAR, which is like the alterations in the nucleus (Figure 3b). Notably, the amount of SIRT1 in mitochondria SIRT1‐SIRT3 colocalization was increased in young but not in aged AAR in response to acute ischemia (Figure 3b). However, the amount of SIRT3 in mitochondria SIRT1–SIRT3 colocalization was augmented in mitochondria in aged but not in young hearts during acute ischemia (Figure 3b). These findings indicate that the deficiency and impaired nuclear shuttling of SIRT1 in aging under acute ischemia cause the increased abundance of mitochondria SIRT1. This adaptive response could maintain the stability of the SIRT1–SIRT3 complex in aged mitochondria during acute ischemia, which could be a protection for SIRT3 and its mediated mitochondria regulation.

We also performed the immunogold double labeling on human cardiomyocytes from the donor's heart to further validate the association between SIRT1 with SIRT3 (Figure 3c). Remarkably, the SIRT1–SIRT3 colocalization was observed in both nucleus and mitochondria of human cardiomyocytes (Figure 3c), suggesting that the association between SIRT1 and SIRT3 could also play an important role in human hearts under physiological and pathological conditions.

To determine whether SIRT1 could interact with SIRT3, we used computational prediction of protein–protein interaction to first analyze the potential SIRT1‐SIRT3 interaction. The docking structure of SIRT1 and SIRT3 with the largest cluster size output from Cluspro 2.0 was shown in Figure 3d. SIRT1 and SIRT3 are colored green and red. The structure shows that an alpha‐helix (from Glu420 to Lys430, highlighted by yellow in Figure 3d) of SIRT1 fitted into the biggest cavity on the SIRT3 surface after binding. Four residues in this alpha‐helix (Glu420, Arg424, Lys427, and Lys430) form very strong electrostatic interactions with residues (Arg158, Glu181, Glu177, and Glu323) of SIRT3, respectively. Besides the helix, another two strong electrostatic interactions (Lys375, Arg446 of SIRT1 with Glu296, Glu325 of SIRT3) on both sides of the alpha‐helix strengthen the binding of the helix of SIRT1 with SIRT3. Our docking results indicated SIRT1 not only fits the shape of the SIRT3 surface but also forms very strong interactions with SIRT3 to stabilize the binding (Figure 3d).

To further determine whether there are alterations in SIRT1–SIRT3 interaction in response to acute ischemia, we performed co‐immunoprecipitation with SIRT1 antibody in the nuclear fraction and mitochondrial fraction from young/aged hearts' whole left ventricle. There was no significant change in the nuclear fraction under acute ischemia. However, acute ischemic stress can trigger upregulation of SIRT1/SIRT3 interaction in the mitochondria of young hearts, which was not observed in the aged hearts (Figure 3e). These data suggest that there is an intracellular nuclear‐shuttling of SIRT1 in response to acute ischemic stress in young hearts, but this nuclear‐shuttling of SIRT1 changes into mitochondria in the aged hearts during acute ischemia. As aging hearts are characterized by mitochondria dysfunction, the increased mitochondrial SIRT1 stabilizes its interaction with SIRT3 and further maintains SIRT3‐mediated mitochondria regulation. These could be one adaptive response to combat the ischemic insults in aging.

### 2.4 SIRT1 deficiency affects SIRT3‐mediated mitochondria substrate metabolic components during acute ischemia and I/R stress

**FIGURE 4:**
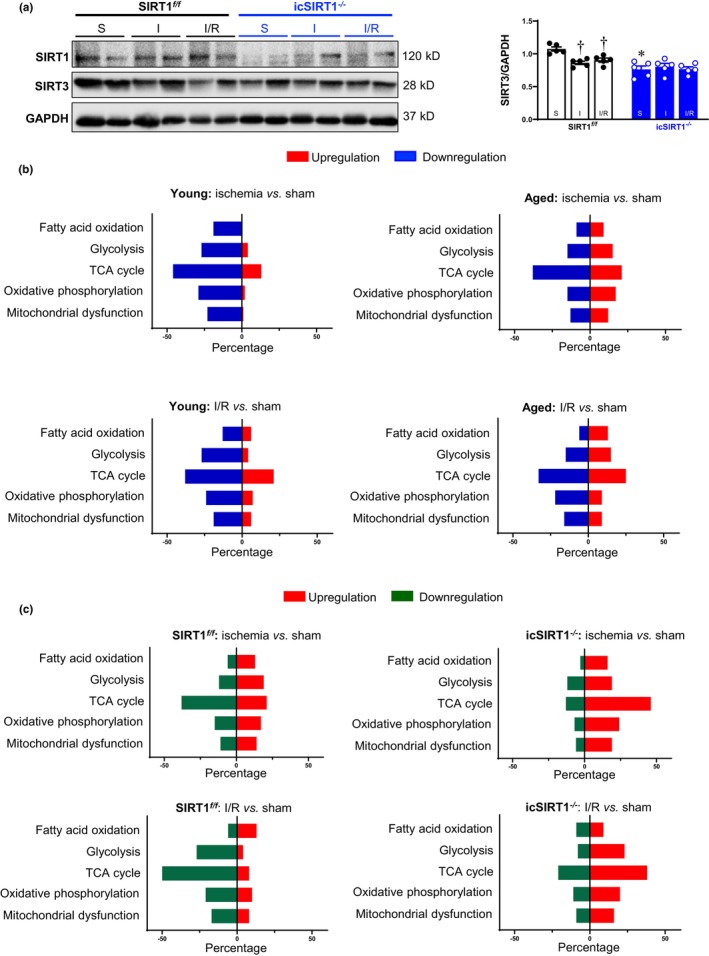
Associate of SIRT1 with SIRT3 in the heart and SIRT3‐associated proteins involved in metabolic regulation in response to acute ischemia and I/R conditions. (a) Western blot analysis of SIRT3 levels in the left ventricle of SIRT1^*f/f*^ and icSIRT1^*−/−*^ male mice hearts under sham, acute ischemia, and I/R conditions. (*N* = 5, values are mean ± SEM from five biological replicates, *p < 0.05 vs. SIRT1^*f/f*^; ^†^ *p* < 0.05 vs. sham, respectively, two‐way ANOVA with Tukey's post hoc test). (b) Ingenuity pathway analysis (IPA) enrichment analysis of the dynamics of SIRT3‐associated proteins in the left ventricle of young (4–6 months)/aged (24–26 months) C57BL/6J male mice hearts in response to acute ischemia and I/R conditions, respectively. Blue bars represent the percentage of genes in the pathway that were downregulated in response to acute ischemia and I/R in young or aged hearts versus sham conditions, respectively. Red bars represent the percentage of genes in the pathway that were upregulated in response to acute ischemia and I/R in young or aged hearts versus sham conditions, respectively. (c) Ingenuity pathway analysis (IPA) enrichment analysis of the dynamics of SIRT3‐associated proteins in the left ventricle of SIRT1^*f/f*^ and icSIRT1^*−/−*^ male mouse hearts in response to acute ischemia and I/R conditions, respectively. Green bars represent the percentage of genes in the pathway that were downregulated in response to acute ischemia and I/R in SIRT1^*f/f*^ or icSIRT1^*−/−*^ hearts versus sham conditions, respectively. Red bars represent the percentage of genes in the pathway that were up‐regulated in response to acute ischemia and I/R in SIRT1^*f/f*^ or icSIRT1^*−/−*^ hearts versus sham conditions, respectively.

To examine whether SIRT1 is critical for the protection of SIRT3‐mediated mitochondria function in the aging heart under physiological and pathological conditions, we determined the protein expression levels of SIRT3 in SIRT1^*f/f*^ and icSIRT1^*−/−*^ mouse hearts with or without acute ischemia and I/R stress. The inducible deletion of cardiomyocyte SIRT1 in adult mice hearts (icSIRT1^−/−^) leads to the downregulation of cardiac SIRT3 (Figure 4a). Acute ischemic stress can reduce SIRT3 levels in SIRT1^*f/f*^ but not in icSIRT1^−/−^ hearts (Figure 4a). Furthermore, the knockdown of SIRT1 in H9C2 cardio myoblasts cells caused SIRT3 expression downregulation (Figure S1A,B). The acetylation of SIRT3 was higher in the aged hearts under acute ischemic stress versus the young group (Figure S1C). Cardiomyocyte SIRT1 deficiency also led to hyperacetylation of SIRT3 (Figure S1D). These observations suggest that SIRT1 could modulate SIRT3 expression and its function in the cardiac mitochondria under physiological and pathological conditions.

To determine the effects of the SIRT1‐SIRT3 interaction on cardiac metabolic homeostasis under physiological and pathological conditions, we performed the SIRT3‐target proteomics analysis of the whole left ventricle under sham operation, acute ischemia, and I/R stress conditions. The results demonstrated that SIRT3 is mainly associated with mitochondrial proteins involved in the respiratory chain complex, tricarboxylic acid cycle (TCA) enzymes, and substrate metabolism‐related proteins (Figure 4b). The comparative proteomics analysis with Integrated pathway (IPA) supplied by QIAGEN was utilized to compare the ischemic stress group with the sham group in young hearts. The results showed that there is a large portion of downregulated SIRT3‐associated proteins involved in the TCA cycle, glycolysis, OXPHOS complex, and fatty acid oxidation upon both acute ischemia and I/R stress in the young hearts (Figure 4b). However, more upregulated SIRT3‐associated proteins showed in aged hearts in response to acute ischemia and I/R stress. Intriguingly, an increased portion of upregulated SIRT3‐targeted proteins also showed in icSIRT1^*−/−*^ group as compared to the SIRT1^*f/f*^ group during acute ischemia and I/R stress (Figure 4c), indicating that cardiac SIRT1 deficiency in aging alters SIRT3‐associated metabolic proteins in response to acute ischemia and I/R stress.

### 2.5 SIRT3 deficiency causes disturbed metabolic reprogramming in response to acute ischemia and I/R stress

**FIGURE 5:**
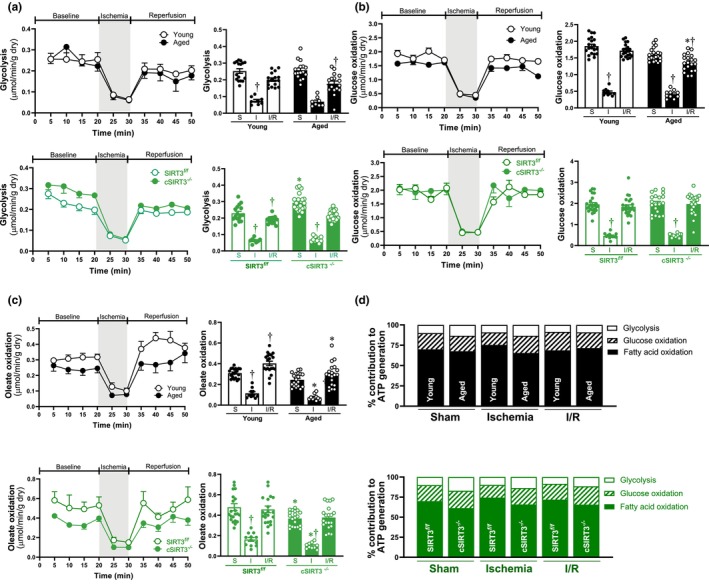
Alterations in substrate metabolism occurred in aged and cSIRT3^*−/−*^ hearts. (a) D‐[5‐^3^H]‐glucose was used in the ex vivo working heart perfusion system to measure glycolysis rate in young, aged, SIRT3^*f/f*^ and cSIRT3^*−/−*^ male mouse hearts subjected to 10 min ischemia and 20 min reperfusion. (*N* = 2–4 time point of each condition, values are mean ± SEM of from four to five biological replicates, *p < 0.05 vs. young, SIRT3^*f/f*^, respectively: ^†^ *p* < 0.05 vs. sham, respectively, two‐way ANOVA with Tukey's post hoc test). (b) Glucose oxidation was analyzed by measuring [^14^C]‐glucose incorporation into ^14^CO_2_ in the ex vivo working heart perfusion system of young, aged, SIRT3^*f/f*^ and cSIRT3^−/−^ male mouse hearts subjected to 10 min of ischemia and 20 min of reperfusion. (*N* = 2–4 time point of each condition, values are mean ± SEM of from four to five biological replicates, *p < 0.05 vs. young, SIRT3^*f/f*^, respectively: ^†^ *p* < 0.05 vs. sham, respectively, two‐way ANOVA with Tukey's post hoc test). (c) The oleate oxidation was analyzed by measuring the incorporation of [9,10‐^3^H] oleate into ^3^H_2_O. (*N* = 2–4 time point of each condition, values are mean ± SEM of from four to five biological replicates, *p < 0.05 vs. young, SIRT3^*f/f*^, respectively: ^†^ *p* < 0.05 vs. sham, respectively, two‐way ANOVA with Tukey's post hoc test). (d) The relative percentage of ATP production calculated from glycolysis, glucose, and oleate oxidation in young, aged, SIRT3^*f/f*^, and cSIRT3^−/−^ hearts.

The ex vivo working heart system model was used to measure the substrate metabolism in young/aged wild‐type and SIRT3^*f/f*^/cSIRT3^*−/−*^ mouse hearts under basal perfusion conditions, acute ischemia, and I/R stress. Glucose oxidation was measured by the amount of [^14^C] glucose metabolism into ^14^CO_2_ in the ex vivo working hearts. Fatty acid oxidation was measured by the incorporation of [9,10‐^3^H_2_O] oleate into ^3^H_2_O. The results demonstrated that acute ischemic stress led to a decreased glycolysis in both young and aged wild‐type hearts (Figure 5a, upper panel); however, the glycolysis rate could not recover after I/R was impaired in the aged hearts versus young hearts (Figure 5a, upper panel). Cardiomyocyte SIRT3 knockout (cSIRT3^−/−^) versus SIRT3^*f/f*^ did not change glycolysis under basal, acute ischemia and I/R conditions (Figure 5a, lower panel). The glucose oxidation determination showed that acute ischemia reduced the rate of glucose oxidation in both young and aged hearts (Figure 5b, upper panel), and glucose oxidation was impaired in aged versus young hearts after I/R treatment (Figure 5b, upper panel). cSIRT3^−/−^ also did not alter glucose oxidation of the ex vivo working perfusion hearts under basal, acute ischemia and I/R conditions versus SIRT3^*f/f*^ mice (Figure 5b, lower panels). The oleate oxidation measurement results demonstrated that acute ischemia caused a decreased rate of oleate oxidation in both young and aged hearts (Figure 5c, upper panel). There was an increase in oleate oxidation during I/R in young hearts but not in aged hearts (Figure 5c, upper panel). cSIRT3^−/−^ showed impaired oleate oxidation under basal, acute ischemia and I/R conditions compared to SIRT3^*f/f*^ (Figure 5c, lower panel). The relative ATP production from glycolysis, glucose oxidation, and oleate oxidation showed that age‐related SIRT3 deficiency plays a dominant role in metabolic remodeling during acute ischemic stress (Figure 5d). In conclusion, our results indicated that SIRT3 is critical to maintain fatty acid metabolism during acute ischemia and I/R stress in the heart, and important to mitochondrial oxidative phosphorylation during acute ischemia. Age‐related cardiac SIRT3 deficiency could be a factor leading to maladaptive metabolic remodeling in the aging heart.

### 2.6 SIRT1 is critical for transcriptional control of fatty acid oxidative enzymes mediated by PGC‐1α/PPARα in response to acute ischemic and I/R stress

**FIGURE 6:**
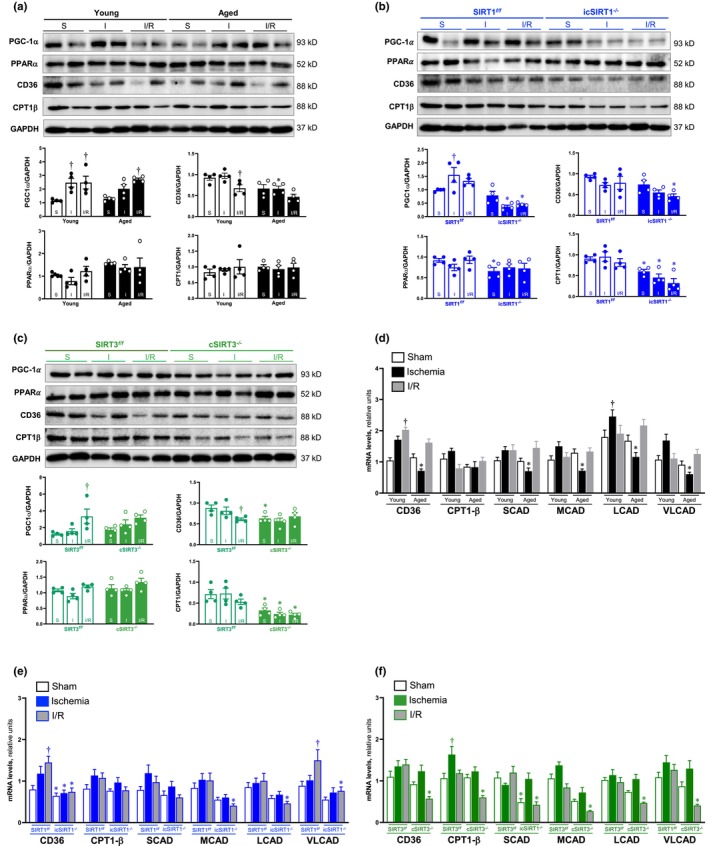
The role of SIRT1 and SIRT3 in regulating fatty acid metabolism mediated by PGC‐1α/PPARα during acute ischemia and I/R stress. (a), (b) and (c) Western blot analysis of PGC‐1α, PPARα, CD36, and CPT1β in young, aged, SIRT1^*f/f*^, icSIRT1^*−/−*^, SIRT3^*f/f*^, and cSIRT3^*−/−*^ left ventricle of male mice hearts under sham, acute ischemia, and I/R conditions. (*N* = 4, values are mean ± SEM from four biological replicates, *p < 0.05 vs. young, SIRT1^*f/f*^, SIRT3^*f/f*^, respectively; ^†^ *p* < 0.05 vs. sham, respectively, two‐way ANOVA with Tukey's post hoc test). (d), (e) and (f) Real‐time PCR analysis of the mRNA levels of CD36, SCAD, MCAD, LCAD, and VLCAD in young, aged, SIRT1^*f/f*^, icSIRT1^*−/−*^, SIRT3^*f/f*^, and cSIRT3^*−/−*^ left ventricle of male mice hearts under sham, acute ischemia, and I/R conditions. (*N* = 3 technical repeat of each replicate, values are mean ± SEM from at least three biological replicates, *p < 0.05 vs. young, SIRT1^*f/f*^, SIRT3^*f/f*^, respectively; ^†^ *p* < 0.05 vs. sham, respectively, two‐way ANOVA with Tukey's post hoc test).

Nuclear receptors serve as substrates for sirtuins and play a role in substrate metabolism via transcriptional level control. Peroxisome proliferator‐activated receptor alpha (PPARα) and PPAR gamma coactivator 1‐alpha (PGC‐1α) are transcriptional regulators of genes that encode enzymes involved in fatty acid oxidation. To determine the role of SIRT1 and SIRT3 in the control of fatty acid metabolism‐related transcription in nucleus, we measured the expression of nuclear receptors PGC‐1α and PPARα. The immunoblotting data showed PGC‐1α accumulation in both young and aged hearts during I/R stress versus to sham group. Only the expression of CD36 was significantly decreased in aged hearts under acute ischemia compared to the young acute ischemia group (Figure 6a). Compared to SIRT1^*f/f*^ mice, icSIRT1^*−/−*^ showed significant downregulation of PGC‐1α and carnitine palmitoyltransferase 1β (CPT1β) under acute ischemia, while PGC‐1α, CPT1β, and CD36 were reduced upon I/R stress (Figure 6b). I/R stress can trigger PGC‐1α in SIRT3^*f/f*^ hearts but not in cSIRT3^−/−^ hearts. Compared to SIRT3^*f/f*^ mice, cSIRT3^−/−^ showed dramatic downregulation of CD36 only under sham conditions, while significant downregulation of CPT1β during sham, acute ischemia, and I/R conditions. (Figure 6c).

PPARα and its cardiac‐enriched coactivator PGC‐1α play important roles in binding the DNA response elements in target gene promoter regions that are associated with fatty acid oxidation. In young hearts, the mRNA level of CD36 was increased during I/R stress and long‐chain acyl‐CoA dehydrogenase (LCAD) was increased during acute ischemia stress (Figure 6d). Interestingly, in aged hearts, the fatty acid transportation, and oxidation‐related genes (CD36, SCAD, MCAD, LCAD, and VLCAD) were significantly decreased during acute ischemic stress compared to the young group (Figure 6d). The compromised transcriptional regulation s closely related to the limited regulation of PGC‐1α in aged hearts during acute ischemia. icSIRT1^−/−^ heart showed reduced mRNA levels of the CD36, MCAD, LCAD, and VLCAD under I/R stress versus SIRT1^*f/f*^ mice (Figure 6e). Similarly, cSIRT3^−/−^ mice showed a significant decrease in the mRNA levels of CD36, CPT1β, MCAD, LCAD, and VLCAD in response to I/R stress versus SIRT3^*f/f*^ hearts (Figure 6f). These results indicated that the aging heart has a greater alteration in PGC‐1α/PPARα‐related transcriptional regulation of fatty acid transportation and oxidation processes upon acute ischemia. Cardiac SIRT1/SIRT3 deficiency mainly causes the downregulation of transcriptional regulation involved in fatty acid transportation and oxidation processes mediated by PGC‐1α/PPARα in response to I/R stress.

### 2.7 SIRT1 is critical for SIRT3 mediated metabolic adaptive in response to acute ischemic and I/R stress

**FIGURE 7:**
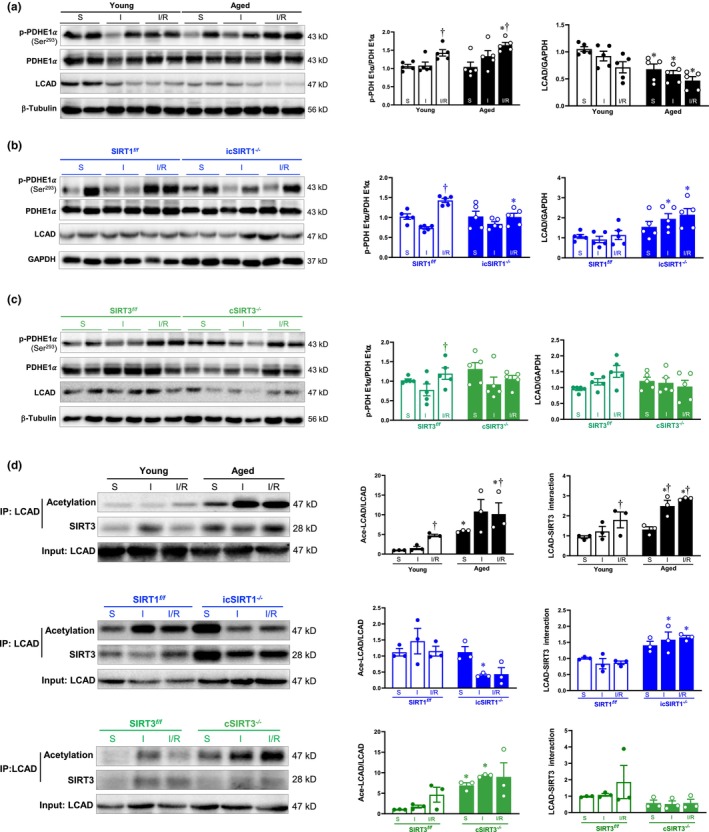
Aged‐related SIRT1 is critical for SIRT3‐mediated fatty acid oxidation during acute ischemia and I/R stress. (a), (b) and (c) Western blot analysis of LCAD and phosphorylation of PDHE1α in young, aged, SIRT1^*f/f*^, icSIRT1^*−/−*^, SIRT3^*f/f*^ and cSIRT3^*−/−*^ left ventricle of male mice hearts under sham, acute ischemia, and I/R conditions. (*N* = 5, values are mean ± SEM from five biological replicates, *p < 0.05 vs. young, SIRT1^*f/f*^, SIRT3^*f/f*^, respectively: ^†^ *p* < 0.05 vs. sham, respectively, two‐way ANOVA with Tukey's post hoc test). (d) Immunoprecipitation analysis of LCAD acetylation and its interaction with SIRT3 in young, aged, SIRT1^*f/f*^, icSIRT1^*−/−*^, SIRT3^*f/f*^ and cSIRT3^−/−^ male mice hearts under sham, acute ischemia, and I/R conditions. (*N* = 3, values are mean ± SEM from three biological replicates, *p < 0.05 vs. young, SIRT1^*f/f*^, SIRT3^*f/f*^, respectively: ^†^ *p* < 0.05 vs. sham, respectively, two‐way ANOVA with Tukey's post hoc test).

Based on the above‐mentioned findings, we aim to explore the SIRT3‐related metabolic target involved in SIRT1‐mediated mitochondria protection. Pyruvate dehydrogenase E1α (PDHE1α) is the critical enzyme deacetylated by SIRT3 that controls the rate of glucose oxidation. We then detected their expression, acetylation, and their interaction with SIRT3 in response to acute ischemia and I/R stress in the heart. To explore whether SIRT1 modulates the activity of the pyruvate dehydrogenase (PDH) via SIRT3, we detected phosphorylated PDHE1α (Ser^293^) acts as an inhibitor on PDH. Interestingly, phosphorylated PDHE1α (Ser^293^) was significantly increased during I/R in young hearts (Figure 7a), which was further exacerbated in aged hearts during I/R (Figure 7a). The increased phosphorylated PDHE1α (Ser^293^) level was also observed in SIRT1^*f/f*^ hearts, which was significantly reduced in icSIRT1^*−/−*^ hearts in response to I/R stress (Figure 7b). Similarly, the increased phosphorylated PDHE1α (Ser^293^) level was observed in SIRT3^*f/f*^ hearts but not in cSIRT3^*−/−*^ hearts in response to I/R stress (Figure 7c). It suggests that the absence of cardiac SIRT1 and SIRT3 could activate PDHE1α during I/R stress, while their deficiency is not associated with the dramatic reduction of activated PDHE1α in aging during I/R. We then aim to know whether the loss of SIRT1‐induced PDHE1α activation is mediated by SIRT3. The hyperacetylation of PDHE1α occurred in the aged hearts compared to the young hearts under both sham, acute ischemia and I/R conditions (Figure S1E). Ischemic stress can increase the PDHE1α‐SIRT3 interaction as an adaptive response in the young acute ischemia group, while this interaction was higher in the aged sham and I/R group except for acute ischemia condition versus the young group, respectively (Figure S1E). However, the hyperacetylation of PDHE1α and PDHE1α‐SIRT3 interaction was not significantly altered between SIRT1^*f/f*^ and icSIRT1^*−/−*^ hearts during both sham, acute ischemia and I/R conditions (Figure S1F). These findings suggest that the regulation of cardiac glucose oxidation via SIRT3‐PDHE1α in the heart may be independent of SIRT1.

LCAD is the critical fatty acid oxidation‐related enzyme, which is also the target of SIRT3 deacetylation. The decreased expression of LCAD was observed in the aged hearts compared to the young hearts under both sham, acute ischemia, and I/R conditions (Figure 7a). Interestingly, an increased LCAD level was observed in icSIRT1^*−/−*^ hearts but not in SIRT1^*f/f*^ hearts in response to acute ischemia and I/R stress (Figure 7b), suggests that SIRT1 is important for LCAD to maintain metabolic homeostasis in response to acute ischemia and I/R stress. We further performed the immunoprecipitation with the LCAD antibody to determine the acetylation status of cardiac LCAD in aging with both sham, acute ischemia, and I/R operations. The results demonstrated that there are hyperacetylation of LCAD occurred in the aged hearts during sham and I/R conditions versus young hearts, respectively (Figure 7d, upper panel). Acute ischemic and I/R stress can trigger the association between LCAD and SIRT3 and acts as an adaptive response to increase SIRT3‐mediated LCAD activation (Figure 7d, upper panel). The icSIRT1^−/−^ hearts showed aging‐like higher levels of LCAD acetylation under sham operation versus SIRT1^*f/f*^ hearts (Figure 7d, middle panel). Intriguingly, acute ischemia and I/R stress increased LCAD acetylation in SIRT1^*f/f*^ hearts but decreased in icSIRT1^−/−^ hearts (Figure 7d, middle panel). That could be due to the compensatory augmented associated with SIRT3 in icSIRT1^−/−^ and the acute ischemia and I/R stress‐mediated activation of SIRT3. However, the cSIRT3^*−/−*^ hearts showed LCAD hyperacetylation under sham operation and exacerbated in response to acute ischemia stress versus SIRT3^*f/f*^ hearts (Figure 7d, lower panel). These data indicated that SIRT1 is critical for SIRT3‐mediated LCAD activity to regulate fatty acid metabolism in cardiac mitochondria under both physiological and pathological conditions.

## 3 DISCUSSION

In this study, we showed an adaptive response regarding the SIRT1‐SIRT3 subcellular location and interaction in aged hearts during acute ischemia stress. In young hearts, SIRT1 is activated by acute ischemia stress and triggers its nuclear shuttling, and results in the enhanced SIRT1‐SIRT3 colocalization in the nucleus. However, since aged hearts are characterized by the mitochondria dysfunction (Lopez‐Otin et al., 2023), SIRT1 changed to translocate into mitochondria in aged hearts during acute ischemia stress. Increased mitochondria SIRT1 recruit more mitochondria SIRT3 to enhance their interaction, acting as adaptive protection for SIRT3 in the aging hearts from further mitochondria dysfunction induced by acute ischemia. The present study evaluated the nuclear and mitochondrial SIRT1/SIRT3 network and pointed out that enhancing mitochondrial sirtuins function is more efficient to protect aging hearts from ischemic insults.

Ischemic heart disease, characterized by constriction in the coronary blood vessel, is known to have a higher morbidity and mortality rate in the elderly population (Dong et al., 2020). Both at clinical and experimental levels, aged hearts are more sensitive to ischemic insults and sustain greater damage during acute ischemia and I/R stress (Wu et al., 2018). The histological and metabolic changes due to senescence are known to be a fundamental factor promoting age‐related changes in the heart. In this study, we have demonstrated that SIRT1 and SIRT3 deficiency aggravates age‐related changes in senescence signaling. In particular, the hallmark of cardiac aging with increased mitochondrial morphology alterations was prominent in SIRT1 and SIRT3 deficiency. Extensive analyses of SIRT3‐mediated proteomics and cardiac metabolic‐related functional evaluation further demonstrated the role of this dynamic SIRT1‐SIRT3 control in preventing greater damage in aging hearts under acute ischemia and I/R stress.

SIRT1 and SIRT3, as powerful anti‐aging regulators, play an important role in protecting hearts from I/R injury. Our lab previously reported that the protein expression level of SIRT1 reduces with aging in hearts and its activity is also limited due to the reduction of NAD^+^ level in aged hearts during myocardial ischemia stress (Tong et al., 2013). Furthermore, SIRT1 administration in aged hearts via AAV delivery increased the tolerance of aged hearts to I/R injury (Wang et al., 2018). The protein expression and activity of SIRT3 are also downregulated with cardiac aging as a result of the decreased NAD^+^ levels (Parodi‐Rullan et al., 2018). The deficiency of SIRT3 in aged hearts increases their tolerance to ischemic insults and I/R injury with increased cardiac reactive oxygen species (ROS) level (Parodi‐Rullan et al., 2017). However, the comprehensive mechanism of how SIRT1 and SIRT3 protect the aged hearts from greater damage upon acute ischemia and I/R stress remains unclear. The study first demonstrated that age‐related deficiency of SIRT1 and SIRT3 changed the cardiomyocyte physiological structure and aggravated cardiac dysfunction after acute ischemic stress and failed recovery through reperfusion operation.

Due to its crucial role in cellular functions, mitochondrial dysfunction has long been considered a major factor in the development of the aging heart (Lopez‐Otin et al., 2023). Previous studies showed that the three populations of cardiac mitochondria possess distinct functional differences (Hollander et al., 2014). Here, we found that age‐related deficiency of SIRT1 and SIRT3 showed more severe damage in all the SSM, IFM and PNM after the acute ischemic operation in the AAR. Notably, only IFM were significantly damaged after I/R stress in the aged AAR verse young group. However, the absence of cardiac SIRT1 seems to have a greater effect on PNM during I/R treatment, while the loss of cardiac SIRT3 seems to have a greater effect on SSM after I/R stress. These data suggest that SIRT1/SIRT3 could work as a complex during acute ischemia, and may control distinct recovery processes during the reperfusion period.

Previous studies revealed age‐related mitochondrial alterations in oxidative phosphorylation (OXPHOS) and ROS production appeared to be limited predominantly to the IFM subpopulation (Hollander et al., 2014). We have demonstrated that aged‐related SIRT1/SIRT3 deficiency impaired cardiomyocyte contractility and is associated with changes in mitochondrial dynamics, OXPHOS and redox homeostasis (Zhang et al., 2021). However, the subcellular localization, activity, and co‐regulatory network of cardiac SIRT1/SIRT3 in aging are still uncertain during acute ischemia and I/R stress. We first confirmed the nuclear translocation and activation of SIRT1 during acute ischemic stress conditions in young hearts and its weakening in aged hearts, which is consistent with our previous study (Tong et al., 2013). In addition, we also found enhanced SIRT1‐SIRT3 colocalization in the nucleus of young hearts at that time. However, SIRT1 changed to translocate into mitochondria in aged hearts' IFM during acute ischemia stress. Increased mitochondria SIRT1 recruit more mitochondria SIRT3 to enhance their interaction. These results suggest that the dynamic SIRT1/SIRT3 subcellular distribution could be protection for the mitochondria in aging hearts from further impairment induced by acute ischemia.

Interestingly, it has been recognized that liver SIRT1 mediated the SIRT3 activity via deacetylation during the obesity and aging process (Kwon et al., 2017). We found that the cardiac deletion of SIRT1 caused the downregulation of SIRT3, which suggests that SIRT1 is a critical regulator for SIRT3 activity under physiological or pathological conditions. We then try to underline the mechanism of how the interaction between SIRT1 and SIRT3 protects hearts from ischemic insults in cardiac aging. The proteomics analysis of SIRT3‐associated proteins in the heart revealed that SIRT3 is closely associated with target proteins involved in the tricarboxylic acid (TCA) cycle, glycolysis, OXPHOS complex, and fatty acid oxidation. Acute ischemia and I/R stress trigger a larger portion of downregulation of SIRT3‐related proteins in young hearts, while increasing the portion of upregulated SIRT3‐related proteins in aged hearts. Intriguingly, the deletion of cardiomyocyte SIRT1 showed similarly greater upregulated SIRT3‐associated proteins in response to acute ischemia and I/R stress. Thus, SIRT1 plays a role in modulating SIRT3‐mediated mitochondrial function and substrate metabolism to adapt to myocardial acute ischemia and I/R stress.

Through an isolated working heart system, the oleate was selected as a fatty acid substrate in the ex vivo heart perfusion as palmitate can have potentially toxic effects on the heart under stress conditions (Russell et al., 2004). In the present study, we found that age‐related SIRT3 deficiency is critical to fatty acid metabolic disorder during acute ischemia and I/R stress in the heart. Alterations in transcriptional and posttranslational control of substrate metabolic enzymes are the primary potential role contributing to the ischemia‐induced shift in mitochondria metabolism in the aged heart. PPARα, as a nutritional sensor, is expressed highly in tissues with high fatty acid oxidation rates such as the liver, heart, and kidney, allowing adaptation of the rates of fatty acid catabolism, and ketone body synthesis under stress conditions (Nakamura et al., 2014). PPARα and its coactivator PGC‐1α are transcriptional regulators of genes involved in mitochondrial β‐oxidation and fatty acid transport (Nakamura et al., 2014). Moreover, CD36 is a high‐affinity receptor for long‐chain fatty acid that facilities the cellular fatty acid uptake (Luiken et al., 2020). Then the long‐chain fatty acids can bind and activate PPARα, thus acting as strong endogenous ligand candidates of PPARα (Nakamura et al., 2014). In this study, we found that the decreased expression of CD36 in the aged hearts during acute ischemia led to the downregulation of endogenous ligands (long‐chain fatty acid), which are the substrate for PPAR‐mediated transcriptional activation of fatty acid metabolism‐related genes. Moreover, the defects of SIRT1 and SIRT3 in icSIRT1^−/−^ and cSIRT3^−/−^ hearts limited the fatty acid transportation to mitochondrial through decreasing CPT1β under sham, acute ischemia and I/R stress. These findings suggest that SIRT1 and SIRT3 are essential for mitochondrial fatty acid metabolism under physiology and pathological conditions. In addition, the deletion of cardiac SIRT1 limited the expression of PGC‐1α and its transcriptional control to fatty acid metabolism‐related genes in response to I/R stress, this may be related to the specific PNM damage in icSIRT1^−/−^ during I/R.

Pyruvate dehydrogenase E1α (PDHE1α) is the major enzyme of the pyruvate dehydrogenase (PDH) complex that transforms pyruvate into acetyl‐CoA that links glycolysis and oxidative phosphorylation (Carrico et al., 2018). Previous studies demonstrated that SIRT3 deacetylates PDHE1α to increase PDHE1α activity (Ozden et al., 2014), and we also confirmed this point. Herein the inactivation of PDHE1α with increased phosphorylation and acetylation of PDHE1α accounts for the impairment of glucose oxidation rate in aged hearts. The SIRT3‐mediated cardiac glucose metabolism through PDH deacetylation is independent of SIRT1, while SIRT1 could influence the activity of PDHE1α through another signaling pathway during I/R stress. Previous mass spectrometry analysis of mitochondrial proteins showed that long‐chain acyl CoA dehydrogenase (LCAD) is hyperacetylated at lysine^42^ in the absence of SIRT3 (Chen et al., 2015). The compromised SIRT3 activity and decreased mitochondria SIRT1–SIRT3 interaction led to aging hearts being more sensitive to acute ischemia. Herein we believed SIRT1 works as an adaptive protection in aging during acute ischemia by increasing its mitochondria distribution and bonding to more SIRT3. The critical regulation of SIRT1 helps maintain SIRT3 deacetylase activity and further activates LCAD at this time. The interaction of SIRT1–SIRT3 in mitochondria helps cardiomyocytes survive from acute ischemic stress via maintain a sufficient energy supply through fatty acid oxidation.

Our present study still has some limitations. First, we choose the AAR in the present study, as AAR is a major determinant of final infarct size and prognosis (Redfors et al., 2012). However, the SIRT1/SIRT3 network needs to be further confirmed in the whole left ventricle and an efficient fraction of nuclear/mitochondria is needed. Second, further analysis of subcellular distribution and interaction of SIRT1 and SIRT3 in PNM and SSM of cardiomyocytes, especially their specific effects and related signaling pathway during I/R stress. The multi‐omics analyses of cardiac IFM, PNM, and SSM are beneficial to understand their division of work under physiological and pathological conditions. Third, more studies are needed to interpret the factors that influence the preference of sirtuins translocation between nuclear and mitochondria. Previous studies have pointed out that the posttranslational modification or mitochondrial/nuclear targeting sequence of sirtuins play important roles in their shuttling (Murugasamy et al., 2022; Tong et al., 2013). Finally, we performed the SIRT1–SIRT3 colocalization in human samples, we advocate studying their role in ischemic heart disease populations to address future clinical usages and therapeutic options.

Taken together, these data revealed that the loss of cardiac SIRT1 and SIRT3 with aging results in an exacerbated cardiac physiological structural and functional deterioration after acute ischemic stress and failed recovery through reperfusion operation. In aged hearts, SIRT1 translocated into mitochondria and recruited more mitochondria SIRT3 to enhance their interaction during acute ischemia stress, acting as adaptive protection for the aging hearts from further mitochondria dysfunction. The increased mitochondria SIRT1–SIRT3 interaction maintains mitochondrial fatty acid metabolism to meet the cardiac energy demand via deacetylation and activation of LCAD under acute ischemia stress. Thus, the mitochondrial SIRT1/SIRT3 network is more efficient to protect aging hearts from ischemic insults and is a promising therapeutic target for numerous aging‐related processes.

## 4 EXPERIMENTAL PROCEDURES

The authors declare that all supporting data are available within the article and the Appendix S1.

Young (3–6 months)/aged (24–26 months) C57BL/6J male mice were supplied from Charles River Laboratories. α‐MHC‐CreER^T2^ (stock number 005657), SIRT1^*f/f*^ mice (stock number 008041), α‐MHC‐Cre (stock number 011038), and SIRT3^*f/f*^ mice (stock number 031201) were purchased from Jackson Laboratory. The SIRT1^*f/f*^ mice were intercrossed with α‐MHC‐CreER^T2^ mice to generate α‐MHC‐CreER^T2^‐SIRT1^*f/f*^ mice. The inducible cardiac‐specific SIRT1 knockout (icSIRT1^−/−^) mice were generated by tamoxifen injection (0.08 mg/g, i.p., 5 days) of α‐MHC‐CreER^T2^‐SIRT1^*f/f*^ (12 weeks old) male mice. Cardiomyocyte‐specific deletion of the SIRT3 (cSIRT3^−/−^) male mice were generated by breeding SIRT3^*f/f*^ mice with transgenic mice that carried an autosomal integrated Cre gene driven by the cardiac‐specific alpha‐myosin heavy chain promoter (α‐MHC‐Cre). SIRT1^*f/f*^ male mice (12 weeks) with tamoxifen injection and SIRT3^*f/f*^ male mice (12 weeks) were used for control groups. All animal protocols in this study were approved by the Institutional Animal Care and Use Committee of the University of South Florida for the care and use of laboratory animals. Human hearts were obtained from donors through the LifeLink at Florida Research Interchange program. The hearts had been considered as prospects for organ donation to the University of South Florida. All tissue handling protocols have been approved by the University of South Florida.

The data, analytic methods, and study materials will be made available to other researchers for the purposes of reproducing the results or replicating the procedures. Expended detailed materials and methods can be founded in the Expanded Materials and Methods in the Appendix S1.

## AUTHOR CONTRIBUTIONS

J. Zhang, H. Wang, F. Cheng, and J. Li designed research, J. Zhang, H. Wang, F. Cheng, and L. Slotabec performed research; J. Zhang, Y. Tan and J. Li analyzed data; and J. Zhang and J. Li wrote the paper.

## CONFLICT OF INTEREST STATEMENT

The authors declare that they have no conflict of interest.

## Supporting information


Appendix S1

## Data Availability

The data that support the findings of this study are available on request from the corresponding author.
