# Supplementary material for: Alterations of SIRT1/SIRT3 subcellular distribution in aging undermine cardiometabolic homeostasis during ischemia and reperfusion
Source: Aging Cell. 2023 Aug 3;22(9):e13930. doi: 10.1111/acel.13930 (PMC10497814; doi:10.1111/acel.13930)
Supplement: Supplementary file 1 — Appendix S1 [file ACEL-22-e13930-s001.pdf]

Alterations of SIRT1/SIRT3 subcellular distribution in aging undermine cardiometabolic homeostasis during ischemia and reperfusion

Jingwen Zhang | Hao Wang | Lily Slotabec | Feng Cheng | Yi Tan | Ji Li

Materials and Methods

In vivo regional ischemia/reperfusion

Male mice of each group were anesthetized, intubated and ventilated as we previously described (Han et al., 2020; Li et al., 2019). Mice were anesthetized with 2%–3% Isoflurane and placed on a heating pad to maintain body temperature at 37°C. After a left lateral thoracotomy, the left anterior descending coronary artery (LAD) was occulated for 30 mins with an 8-0 nylon suture and polyethylene tubing to prevent arterial injury and subsequently reperfused for 6 hours. ECGs were utilized to confirm the ischemic hallmark of the ST-segment elevation during coronary occlusion (AD Instruments, Colorado Springs, CO). At the end of reperfusion, the hearts were excised, and left ventricles were separated before freeze clamping in liquid nitrogen.

Myocardial infarct size measurements

After 6h of reperfusion, mice were anesthetized and ventilated as aforementioned. Non-necrotic tissue in the ischemic region was stained red by TTC (1%, w/v) and the non-ischemic region was stained blue with Evan's blue (1%, w/v). The hearts were left in 4% paraformaldehyde overnight, then fixed, sectioned into 1-2 mm slices, photographed with a Leica microscope (Leica Microsystems), and analyzed with Image J software (National Institutes of Health) (Li et al., 2019; Quan et al., 2017). The myocardial infarct size was calculated as the ratio of the percentage of myocardial necrosis (Infarction) to the ischemic area at risk (AAR).

Myocardial histology

The left ventricular tissue from normal physiological or 30 min ofischemiaand 6h reperfusion was rapidly excised and fixed in 4% buffered paraformaldehyde. Fixed tissue was then paraffinembedded and sectioned and stained with H&E. Slides were thenassessed in a blinded fashion with Keyence BZ-X710. All-in-One Fluor-escence Microscope under 20X objective magnification power.

Subcellular fractionation for nuclear, cytoplasm and mitochondria

Mouse heart left ventricule subcellular fractions were prepared using a cell fractionation kit (Abcam, ab109719, USA) according to the manufacturer's instruction with modifications. The nuclear-cytoplasmic fraction of left ventricule tissue or cells was conducted using the according to the manufacturer's protocol. Tissues were collected in 10 ml of ice-cold PBS and minced with a razor blade. The minced tissue was washed with ice-cold PBS and resuspended following the protocol in cytosolic buffer. The sample was transferred to a prechilled Dounce homogenizer and then incubated for 10 min. The tissue was centrifuged at 12000 g for 10 min twice at 4 °C. The resulting supernatant was the cytoplasm fraction. The pellet was resuspended following the protocol in mitochondrial buffer. After 10-min incubation. The tissue was then centrifuged at 10,000 g for 10 min twice at 4 °C to pellet the nuclear. The supernatant was collected as mitochondrial fraction. All the buffers and solutions were supplemented with protease and phosphatase inhibitors.

Immunoprecipitation and Immunoblotting

Immunoblots and immunoprecipitation were performed as previously describe(Quan et al., 2017). For immunoprecipitation analysis, lysates were mixed with antibodies at 4°C 1 h followed by the addition of 40 μl of Protein A/G PLUS-Agarose (Santa Cruz) at 4°C overnight. Immune complexes were washed three times with lysis buffer and then boiling. Samples were subjected to SDS/PAGE. Equivalent amounts of protein (20 μg per lane) were loaded and separated by 10% SDS-PAGE gels and transferred to polyvinylidene difluoride (PVDF) membranes (Millipore, Bedford, MA). For reprobing, membranes were stripped with Restore Western Blot Stripping Buffer (Thermo Fisher Scientific). Rabbit polyclonal antibodies against SIRT1, LCAD, PPARα, phospho-PDH E1α(phosphoSer-293) and Membrane Fraction WB Cocktail were purchased from Abcam (Cambridge, MA,). Rabbit monoclonal antibody PGC-1α, CPT-1β, SIRT3 were purchased from Cell signaling (Danvers, MA). Mouse polyclonal antibodies against CD36 and PDH E1α from Santa Cruz (San Diego, CA) were purchased and used according to protocols provided by the manufacturer. Image Lab (Bio-rad) was used to quantify the optical density of each band.

Proteomics analysis

Immunoprecipitated proteins with SIRT3 antibody were digested and subjected to mass spectrometric analysis with Thermo Q-exactive-HF mass spectrometer coupled to a Thermo Easy nLC

1200. Output data was searched against Uniprot reviewed Mouse database using Thermo Proteome Discoverer 2.2 software. Max quant analysis was then applied to each group among biological replicates (n=3) and Welch's t-test was performed to determine the z-score cut off between each pair comparison. Furthermore, ingenuity pathway analysis (IPA) powered by QIAGEN was utilized to identify the involved pathways of SIRT3 associated proteins and upstream transcriptional regulators that can explain observed gene expression changes in the dataset.

mRNA analysis by quantitative polymerase chain reaction (qPCR)

Mouse heart left ventriclar tissues were collected for RNA extraction using TRIzol® reagent (Invitrogen). mRNA was reverse transcribed into double-stranded cDNA fragments using the Thermo Script RT-PCR system (Invitrogen), with 1 µg total RNA and 1 µl reverse transcriptase, in accordance with the manufacturer's instructions. qPCR was performed using 10-µl reactions, which contained 10 ng cDNA, 10 nM each for forward and reverse primers, and 10 ng SYBR Green Supermix (Bio-Rad). A thermocycler (QuantStudio 3 RealTime PCR Systems) was used for amplification with the following protocol: 95 °C for 10 min, followed by 40 cycles of 95 °C for 10 s and 60 °C for 45 s. For each target gene, a standard curve was generated, and the starting quantity of mRNA was calculated using Bio-Rad qPCR detection system software. All transcripts were analyzed in duplicate and normalized to 18s-RNA. The delta delta Ct method was used to analyze the results. Primer sequences for qPCR are provided in Supplementary Table.

Immunofluorescence

Isolated hearts were perfused and incubated with 4% paraformaldehyde for 1 h at room temperature. Tissues were obtained from the area at risk (AAR) of mice heart left ventricle, and then transferred to 30% sucrose overnight at 4°C, mounted in Tissue-Tek Optimal Cutting Temperature (OCT) compound (Sakura Finetek, Torrance, CA, USA), snap-frozen in liquid nitrogen, and stored at −80°C overnight. The samples were sliced into 10-µm-thick sections on a cryostat, mounted onto clean glass slides that had been charged by a chromium, potassium, and gelatin solution, thoroughly air-dried, and stored at −80°C until processing. For staining, slides were incubated with 0.3% Triton X-100 in PBS for 15 min at room temperature, washed with PBS, blocked with 10% donkey serum, 5% BSA and 0.3% Triton-X in PBS for 1 h, and then incubated overnight with anti-SIRT1 antibody (Santa Cruz), anti-SIRT3 antibody (Abcam) and anti-Troponin T (Abcam) in a 5% BSA-TBST solution. The bound antibodies were labeled using Alexa Fluor 488-conjugated goat anti-rabbit secondary antibody and Alexa Fluor 594-conjugated goat anti-mouse secondary antibody (Thermo Fisher Scientific). Sections were then stained with

DAPI and covered using Anti-Fade Fluorescence mounting medium (Abcam). Images were captured using an Olympus FV1200 confocal microscope and analyzed by Image J software as previously describe (Shihan, Novo, Le Marchand, Wang, & Duncan, 2021). For measurements of mean fluorescence intensity (MFI) of tissue sections, we used the 'Hyperstack', 'Colorized' and channels' option to independent analyse each fluorescent channels. Once the desired tissue area is circled, click on the 'Analyze' button to select the 'Measure' option. Final MFI = MFI of tisseue sections– MFI of Background. For measurements of MFI of nuclear-localized proteins tissue sections, the original confocal image of a section were split into independent fluorescent channels. Adjusting the green channel to select for nuclei stained for SIRT1/SIRT3 protein by using the 'Threshold' button. Click on the 'Reset' button to get back to the original image and results of MFI quantitation of SIRT1/SIRT3 levels in nuclei.

Fatty acid/glucose oxidation analysis

The working heart system preload and afterload were set at 15cm and 80cm H_2O, respectively. The flow rate was kept at 4 ml/min and change to 1mL/min during ischemia. [9, 10]-^{3}H-oleate (50 mci/L) and ^{14}C-glucose (20 mci/L)–labeled BSA buffer was perfused into the heart via the pulmonary vein and pumped out through the aorta. Non-recycled perfusate that was pumped out from the aorta and outflowed from coronary venous artery and collected every 5 min to test the radioactivity. The fatty acid level was determined by the production of ^3H_2O from [9, 10]-^{3}H-oleate. Metabolized ^3H_2O was separated from [9, 10]-^{3}H-oleate by filtering through anion-exchange resin (Bio-Rad, Hercules, CA, USA). Glucose oxidation was measured and sampled every 5 min by both the metabolized ^{14}CO2 that was dissolved in the perfusate buffer and by the gaseous ^{14}CO2, which was further dissolved in sodium hydroxide. To separate ^{14}CO2 from ^{14}C-glocose, sulfuric acid was added to perfusate samples to release ^{14}CO2. ^{3}H and ^{14}C signals were detected to discriminate metabolic products from fatty acid and glucose, respectively. For calculating the rate of glucose oxidation or oleate oxidation, place the the whole perfuse-heart in an oven set at 50 °C overnight and measure its dry weight firstly. For each time point x express the value of coronary flow CF_x in mL/min. Secondly, determine the ^{14}C or ^{3}H averaged value of specific activity ^{14C}d.p.m.$_{sa}$ or ^{3H}d.p.m.$_{sa}$. Thirdly, average the two disintegration/min (d.p.m.) values measured for the background activity of ^{14C}d.p.m.$_{ba}$ and ^{3H}d.p.m.$_{ba}$. Determine the specific radioactivity of glucose or oleate in d.p.m./μmol ($F_{Glu\ or}$ F_{Ole}), F_{Glu}= ^{14C}d.p.m.$_{sa}$/C_{Glu} (μmol/L)*sample volume (L) or F_{Ole} = ^{3H}d.p.m.$_{sa}$/C_{Ole} (μmol/L)*sample volume (L). Finally, determine for each time point x the rate of production of ^{14}CO2 in d.p.m./min per g dry wt (A_{norm}) or or ^{3}H in

d.p.m./min per g dry wt (B_{norm}), A_{norm}= (^{14C}d.p.m.$_x$-^{14C}d.p.m.$_{ba}$)/ Volx (mL) * CF_x/ per dry wt (g), B_{norm}= (^{3H} d.p.m.$_x$-^{3H} d.p.m.$_{ba}$)/ Volx (mL) * CF_x/ per dry wt (g). The rate of glucose oxidation (GO)= A_{norm}/F_{Glu} and the rate of oleate oxidation (OO)= B_{norm}/F_{Ole}.

Glycolysis analysis

Glycolysis was analysed in the isolated working heart perfusion system by measuring the production of ^3H_2O D-[5-3 H]-glucose. Mice were heparinized (100 units i.p.) 10 min before being anesthetized. Isolated hearts were then retroperfused in the Langendorff perfusion system (Radnoti, Monrovia, CA) with Krebs–Henseleit buffer (KHB) containing 7 mM glucose, 1% BSA, 0.4 mM sodium oleate, 10 µU/mL insulin, and D-[2-3 H]-glucose/D-[5-3 H]-glucose bubbled with 95% O_2/5% CO_2. The whole system was kept at 37°C. For glycolysis measurements, isolated hearts were subjected to 20 min basal perfusion, followed by 10 min global, low-flow ischemia and then 20-min reperfusion. Perfusate was recycled and collected every 5 min to test the radioactivity. Metabolized ^3H_2O was separated from D-[5-3 H]-glucose by filtering through an anion exchange 1-X8 resin (Bio-Rad, Hercules, CA). The rate of glycolysis was calculated by the amount of ^3H_2O production. About 10 mL of scintillation fluid was added to each vial and then mixed well. The radioactive signal was measured on a liquid scintillation counter. The calculation of the glycolysis rate works as the way of calculating oleate oxidation rate. Rates of ATP production from energy metabolism were calculated based on 2 ATP produced for molecule of glucose passing through glycolysis, 30 ATP for each molecule of glucose oxidized, and 105 ATP for each Oleate molecule oxidized.

Transmission electron microscope

The area at risk (AAR) of mice heart left ventricule were rapidly immersed in McDowell's Trump Fixative (Electron Microscopy Science, PA) at 4°C for 48 h. Fixed tissues were trimmed to 1 mm^3 in size, stained with OsO4 for 4 h, dehydrated in a graded ethanol series for 20 min per step (once in 35%, 50%, 70%, and 95% and twice in 100% ethanol), washed twice with acetone for 10 min per wash, washed in a solution of 2:1 acetone: resin for 1 h, 1:1 acetone: resin for 2 h, 1:2 acetone: resin for 2 h, pure resin for 3 h and finally embedded. Semithin sections were stained with 1% toluidine and observed under a light microscope to locate interesting areas for ultrathin sections. After the trim, the block was thin sectioned (90-100 nm thick) and then applied to copper grids and air dried. The grid was loaded in a JEOL1400 transmission electron microscopy (Jeol, Tokyo, Japan). 2 biological replicates with at least 5 technical repeat of each replicate to capture the image at a magnification of 20,000 to obseve the mitochondria morphology

alterations. Final representitave images of mitochondria each group was showed at a magnification of 60,000.

Quantitative Postembedding Immunogold Electron Microscopy

Cardiac left ventricle was perfused with 4% Paraformaldehyde (PFA) and 0.1% glutaraldehyde (GA) fixation solution and tissue were sectioned of the AAR in mice heart left ventricule. For double labeling, the sections were first incubated with anti-SIRT1 antibody followed by goat anti-rabbit coupled to 10 nm colloidal gold. The sections were exposed to formaldehyde vapor at 80°C for 1 h and thereafter incubated with rabbit polyclonal anti-syntaxin-1, followed by anti-SIRT3 antibody coupled to 15 nm colloidal gold. The sections were the fixed 1% glutaraldehyde and examined with JEOL1400 transmission electron microscopy (Jeol, Tokyo, Japan). Electron micrographs from mice were obtained and quantified as number of gold particles/μm^2 of cardiac mitochondria and nuclear compartment. Electron micrographs with gold particle staining were taken for each specimen at a magnification of 100,000 as representative. Positive pictures at a magnification of 20,000 analyzed by automated gold particle quantification system as previously describe (Enger, 2017). After defining parameters and area segmentation by use of a polygon tool, the methods of automatic particle detection based on multi-level thresholding was use in this project.

Protein Docking Analysis

Protein-protein docking was performed to investigate whether SIRT1 could interact with SIRT3. X-ray crystal structures of SIRT1 and SIRT3 were downloaded from the Protein Data Bank (PDB) database. Human SIRT1 (PDB id: 4IG9) and human SIRT3 crystal structure (PDB id: 3GLS) were inputted to the Cluspro 2.0 web server (https://cluspro.bu.edu/) for protein-protein docking. The docking structures of SIRT1-SIRT3 complex were ranked by the cluster size calculated using the defaulted parameters (the balanced model and 70000 rotations) of Cluspro. The docking structures were displayed by the Biovia Discovery Studio software.

Cell Culture and hypoxia and reoxygenation (H/R) treatment

Rat cardiac myoblast H_9C_2 cells (ATCC CRL-1446™) were cultured in high glucose (4.5 g/L) DMEM (Corning Cellgro) supplemented with 10% (v/v) fetal bovine serum (FBS) (Gibco. Life Technologies) and antibiotics (100 U/mL penicillin and 100 μg/mL streptomycin) (ATCC). The cells were maintained in a humidified incubator with 95% air and 5% CO2 at 37°C. H_9C_2 were placed in a nitrogen chamber containing 95% N2/5% CO2 for 12 hours to induce hypoxia and then reoxygenate for 6 hours under normal atmospheric oxygen levels.

Transient transfection with small interfering RNA (siRNA) and cycloheximide chase assays

To knockdown the endogenous SIRT1, H_9C_2 cells were transiently transfected with 100 nM mouse siRNAs targeting SIRT1 (Ambion) or with the non-silencing control siRNA (scram) (Ambion) using Lipofectamine™ RNAiMAX (Invitrogen) in culture medium without antibiotics according to the manufacturer's recommendations. For cycloheximide chase assays, cells were treated with or without 100 mg/mL of cycloheximde (*Beyotime*) for 30, 60, 90, and 120 min, and then lysaed for immunoblotting.

Statistics analysis

The analysis results of cardiac function, superoxide accumulation, and immunoblotting were expressed as means ± standard error of the means (SEM). Welch's t-test, one-way ANOVA with Tukey's test and Kruskal-Wallis test were used to were used to perform the comparison of the statistics among a set of samples with Prism 8.0 (GraphPad Software). $P<0.05$ was considered a significant difference.

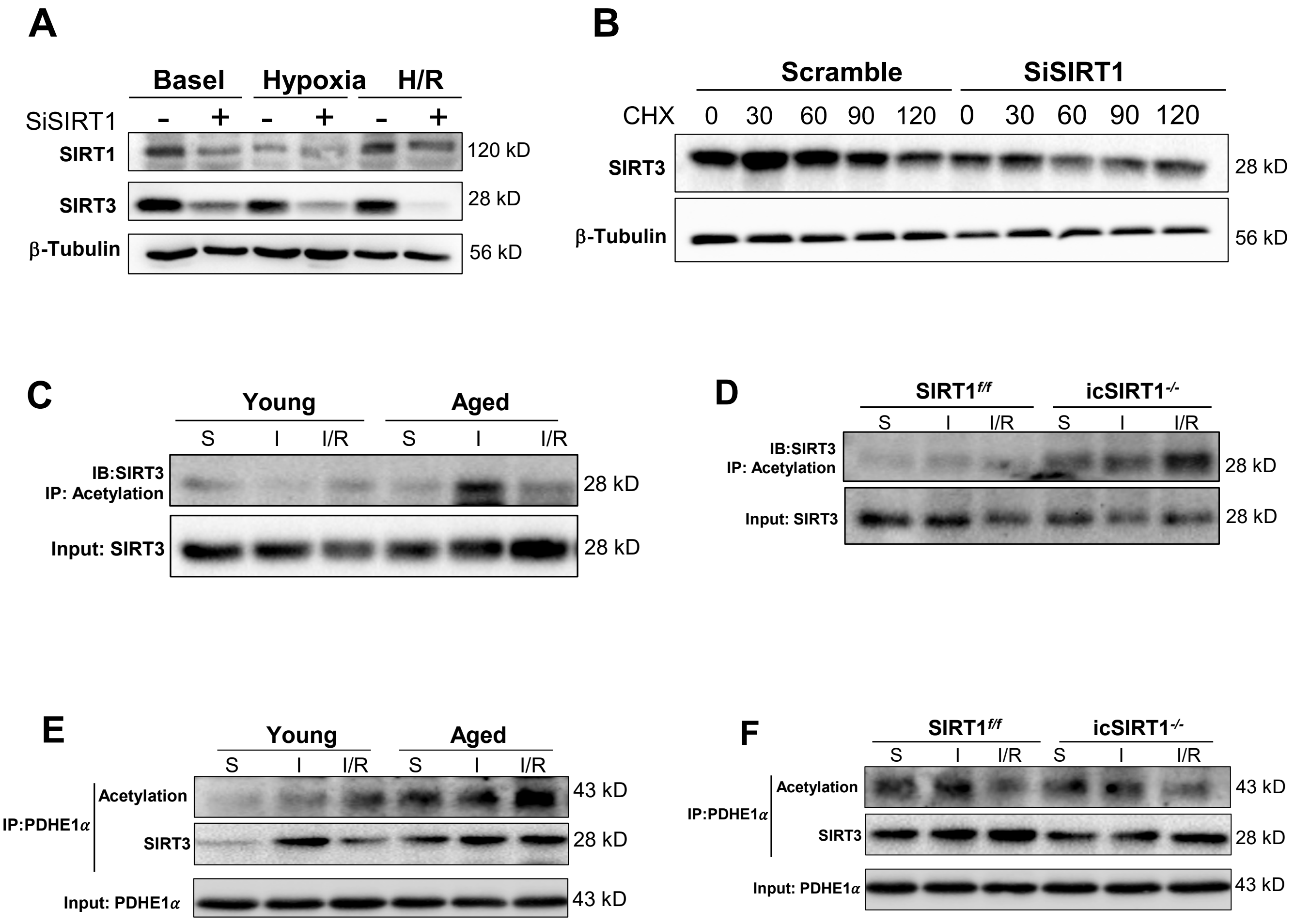

Suppl. Figure 1. SIRT1 is critical for the acetylation, stability and activity SIRT3-mediated in response to I/R stress. (A), Western blot analysis of SIRT3 expression after Scramble and SiRNA treatment in H9C2 cardiomyoblast cells under basal, hypoxia, and hypoxia/reoxygenation (H/R) stress conditions. (B) Half-life of SIRT3 was assessed using cycloheximide (CHX, 100 μg/mL) in H9C2 cells transfected with SiSIRT1. (C) and (D) Immunoprecipitation analysis of SIRT3 acetylation and its interaction with SIRT3 in young and aged (C) and $SIRT1^{f/f}$ and $icSIRT1^{-/-}$ (D) male mice hearts left ventricle under sham, ischemia, and I/R stress conditions. (E) and (F) Immunoprecipitation analysis of SIRT3 acetylation and its interaction with SIRT3 in young and aged (E) and $SIRT1^{f/f}$ and $icSIRT1^{-/-}$ (F) male mice hearts left ventricle under sham, ischemia, and I/R stress conditions.

Suppl Table 1. Echocardiographic measurements of mouse hearts functions under either sham or ischemia (30 min)/reperfusion (6 h) (I/R) conditions

Group	Young		Aged		SIRT1$^{f/f}$		icSIRT1$^{-/-}$		SIRT3$^{f/f}$		cSIRT3$^{-/-}$	
	**Sham**	**I/R**	**Sham**	**I/R**	**Sham**	**I/R**	**Sham**	**I/R**	**Sham**	**I/R**	**Sham**	**I/R**
**HR**	440.19 ± 22.7	412.25 ± 35.94	393.73 ± 20.62	416.72 ± 53.95	403.1 ± 30.64	396.49 ± 58.39	458.28 ± 36.84	429.78 ± 19.10	421.76 ± 53.94	409.11 ± 51.65	430.02 ± 45.93	403.42 ± 49.31
**CO**	18.62 ± 5.67	9.15 ± 2.47*	17.31 ± 1.80	9.31 ± 1.93*	15.68 ± 2.29	12.1 ± 3.48	16.89 ± 1.77†	11.46 ± 2.10*†	15.84 ± 2.18	13.19 ± 4.24*	16.09 ± 2.99	9.05 ± 5.45*†
**LV Mass**	127.1 ± 24.25	110.53 ± 22.04	181.83 ± 36.45	166.22 ± 24.58	131.99 ± 40.21	148.56 ± 67.84	208.97 ± 74.22	154.2 ± 20.88	124.56 ± 8.82	122.99 ± 10.84	120.95 ± 28.07	121.54 ± 25.07
**SV**	42.14 ± 11.76	22.48 ± 6.88*	44.14 ± 5.78	22.67 ± 5.62*	39.71 ± 3.03	28.41 ± 3.52*	37.04 ± 4.70	26.67 ± 4.84*	37.74 ± 4.59	29.26 ± 3.53*	37.60 ± 6.95	19.93 ± 10.15*
**LVAW;s**	1.4 ± 0.15	1.08 ± 0.28	1.50 ± 0.15	1.27 ± 0.25	1.41 ± 0.15	1.21 ± 0.36	1.55 ± 0.48	1.14 ± 0.29	1.18 ± 0.17	1.14 ± 0.12	1.31 ± 0.09	1.34 ± 0.16
**LVAW;d**	0.92 ± 0.06	0.86 ± 0.13	1.02 ± 0.19	1.03 ± 0.24	0.95 ± 0.17	0.91 ± 0.31	1.14 ± 0.36	0.97 ± 0.23	0.78 ± 0.09	0.87 ± 0.08	0.82 ± 0.12	1.09 ± 0.14
**LVPW;s**	1.41 ± 0.15	1.06 ± 0.18	1.48 ± 0.58	1.4 ± 0.38	1.47 ± 0.10	1.27 ± 0.52	1.54 ± 0.27	1.16 ± 0.22	1.22 ± 0.06	1.10 ± 0.14	1.28 ± 0.21	1.17 ± 0.3
**LVPW;d**	0.92 ± 0.23	0.85 ± 0.21	1.13 ± 0.40	1.15 ± 0.3	0.96 ± 0.22	1.01 ± 0.43	1.23 ± 0.23	0.94 ± 0.24	0.90 ± 0.17	0.85 ± 0.12	0.99 ± 0.22	0.92 ± 0.30
**Volume;s**	22.81 ± 3.89	35.66 ± 18.87	28.75 ± 11.74	39.7 ± 14.68	22.98 ± 1.28	35.07 ± 10.25	28.31 ± 14.4	42.57 ± 6.72	28.72 ± 4.06	33.81 ± 7.25	19.20 ± 3.00	26.27 ± 10.14
**Volume;d**	59.95 ± 16.64	58.14 ± 23.12	72.88 ± 915.11	62.37 ± 17.57	56.02 ± 4.40	65.14 ± 10.26	72.02 ± 24.72	74.24 ± 14.14	69.78 ± 11.76	65.57 ± 10.90	56.79 ± 9.71	47.86 ± 20.92
**Diameter;s**	2.26 ± 0.31	2.93 ± 0.72	2.72 ± 0.49	3.12 ± 0.47	2.18 ± 0.28	2.97 ± 0.38	2.83 ± 0.93	3.39 ± 0.33	2.87 ± 0.34	2.94 ± 0.26	2.35 ± 0.15	2.63 ± 0.41
**Diameter;d**	3.72 ± 0.46	3.64 ± 0.63	4.05 ± 0.36	3.78 ± 0.44	3.64 ± 0.12	3.87 ± 0.27	4.00 ± 0.61	4.08 ± 0.34	3.98 ± 0.28	3.88 ± 0.27	3.65 ± 0.26	3.34 ± 0.65

Note: HR, Heart rate; **CO,** Cardiac output; **SV,** Stroke volume; **IVAW;s**, Left ventricular end-systolic anterior wall thickness (systolic); **IVAW;d,** Left ventricular end-diastolic anterior wall thickness (diastolic); **IVCT,** Isovolumic contraction time; **LVPW;s**, Left ventricular posterior wall (systolic); **LVPW;d,** Left ventricular posterior wall (diastolic); **Volume;s**, Left ventricular end-systolic volume (systolic); **Volum;s**, Left ventricular end-diastolic volume (diastolic); **Diameter;s,** left ventricular internal dimension at end systole; **Diameter;s,** left ventricular internal dimension at end diastole.

N=6, Values are expressed as mean ± SD. *p<0.05 *vs.* sham,respectively; †p<0.05 *vs.* young I/R, SIRT1$^{f/f}$ I/R, SIRT3$^{f/f}$ I/R, respectively.

Suppl Table 2. Hemodynamic parameters of isolated working heart perfusion system.

Group	Young		Aged		$SIRT3^{f/f}$		$cSIRT3^{-/-}$	
	Basal	I/R	Basal	I/R	Basal	I/R	Basal	I/R
HR (min^{-1})	293.33 ± 36.30	228.33 ± 30.66	254.33 ± 36.23	196.33 ± 12.06	312.00 ± 15.87	241.00 ± 11.53	281.67 ± 12.74	221.00 ± 6.08
CO (ml min^{-1})	3.53 ± 0.19	3.26 ± 0.28	3.44 ± 0.13	2.99 ± 0.05*†	3.47 ± 0.38	3.12 ± 0.09	3.22 ± 0.09	2.57 ± 0.25*†
LVDP (mmHg)	21.17 ± 6.70	20.62 ± 7.40	17.87 ± 2.93	13.15 ± 3.47	24.57 ± 0.94	23.30 ± 2.46	22.52 ± 5.60	16.48 ± 2.57

Note: HR: heart rate; **CO**: cardiac output; **LVDP**: left ventricle developed pressure. N=3, Values are expressed as mean ± SD. *p<0.05 *vs.* basal, respectively; †p<0.05 *vs.* young I/R, $SIRT1^{f/f}$ I/R, $SIRT3^{f/f}$ I/R, respectively.

Suppl Table 3. Real-time PCR primer list.

Gene		Sequence(5'-3')
**CD36**	Forward	TGG CCT TAC TTG GGA TTG G
	Reverse	CCA GTG TAT ATG TAG GCT CAT CCA
**CPT1β**	Forward	CCT CCG AAA AGC ACC AAA AC
	Reverse	GCT CCA GGG TTC AGA AAG TAC
**SCAD**	Forward	ACC AAA GCT TGG ATC ACC AAC TCC
	Reverse	AAC CAG GAA GGC ACT GAT ACC CTT
**MCAD**	Forward	TGT TAA TCG GTG AAG GAG CAG
	Reverse	CTA TCC AGG GCA TAC TTC GTG
**LCAD**	Forward	GGT GGA AAA CGG AAT GAA AGG
	Reverse	GGC AAT CGG ACA TCT TCA AAG
**VLCAD**	Forward	GGC CAA GCT GGT GAA ACA CAA GAA
	Reverse	ACA GAA CCA CCA CCA TGG CAT AGA
**18s-RNA**	Forward	TTT CGC TCT GGT TCG TCT TG
	Reverse	GGA TAC CGC AGC TAG GAA TAA T

References

Enger, R. (2017). Automated gold particle quantification of immunogold labeled micrographs. *J Neurosci Methods, 286*, 31-37. doi:10.1016/j.jneumeth.2017.05.018

Han, Y., Sun, W., Ren, D., Zhang, J., He, Z., Fedorova, J., . . . Li, J. (2020). SIRT1 agonism modulates cardiac NLRP3 inflammasome through pyruvate dehydrogenase during ischemia and reperfusion. *Redox Biol, 34*, 101538. doi:10.1016/j.redox.2020.101538

Li, X., Liu, J., Hu, H., Lu, S., Lu, Q., Quan, N., . . . Li, J. (2019). Dichloroacetate Ameliorates Cardiac Dysfunction Caused by Ischemic Insults Through AMPK Signal Pathway-Not Only Shifts Metabolism. *Toxicol Sci, 167*(2), 604-617. doi:10.1093/toxsci/kfy272

Quan, N., Sun, W., Wang, L., Chen, X., Bogan, J. S., Zhou, X., . . . Li, J. (2017). Sestrin2 prevents age-related intolerance to ischemia and reperfusion injury by modulating substrate metabolism. *FASEB J, 31*(9), 4153-4167. doi:10.1096/fj.201700063R

Shihan, M. H., Novo, S. G., Le Marchand, S. J., Wang, Y., & Duncan, M. K. (2021). A simple method for quantitating confocal fluorescent images. *Biochem Biophys Rep, 25*, 100916. doi:10.1016/j.bbrep.2021.100916
